# Benzothiadiazole versus Thiazolobenzotriazole: A Structural Study of Electron Acceptors in Solution‐Processable Organic Semiconductors

**DOI:** 10.1002/asia.202200768

**Published:** 2022-09-30

**Authors:** Nanami Watanabe, Waner He, Naoya Nozaki, Hidetoshi Matsumoto, Tsuyoshi Michinobu

**Affiliations:** ^1^ Department of Materials Science and Engineering Tokyo Institute of Technology 2–12-1 Ookayama, Meguro-ku Tokyo 152–8552 Japan

**Keywords:** contact resistance, electron acceptors, n-channel transistors, organic semiconductors

## Abstract

Despite the rapid progress of organic electronics, developing high‐performance n‐type organic semiconductors is still challenging. Donor‐acceptor (D‐A) type conjugated structures have been an effective molecular design strategy to achieve chemically‐stable semiconductors and the appropriate choice of the acceptor units determines the electronic properties and device performances. We have now synthesized two types of A_1_‐D‐A_2_‐D‐A_1_ type conjugated molecules, namely, NDI‐BTT‐NDI and NDI‐TBZT‐NDI, with different central acceptor units. In order to investigate the effects of the central acceptor units on the charge‐transporting properties, organic field‐effect transistors (OFETs) were fabricated. NDI‐TBZT‐NDI had shallower HOMO and deeper LUMO levels than NDI‐BTT‐NDI. Hence, the facilitated charge injection resulted in ambipolar transistor performances with the optimized hole and electron mobilities of 0.00134 and 0.151 cm^2^ V^−1^ s^−1^, respectively. In contrast, NDI‐BTT‐NDI displayed only an n‐channel OFET performance with the electron mobility of 0.0288 cm^2^ V^−1^ s^−1^. In addition, the device based on NDI‐TBZT‐NDI showed a superior air stability to that based on NDI‐BTT‐NDI. The difference in these OFET performances was reasonably explained by the contact resistance and film morphology. Overall, this study demonstrated that the TBZ acceptor is a promising building block to create n‐type organic semiconductors.

## Graphical abstract

**Acceptor structures of** benzothiadiazole (BT) and thiazolobenzotriazole (TBZ) are compared for use in organic field‐effect transistors. The TBZ‐based semiconductor showed one order of magnitude higher electron mobility than the BT‐based semiconductor. This is due to its charge injection efficiency and film forming capabilities.

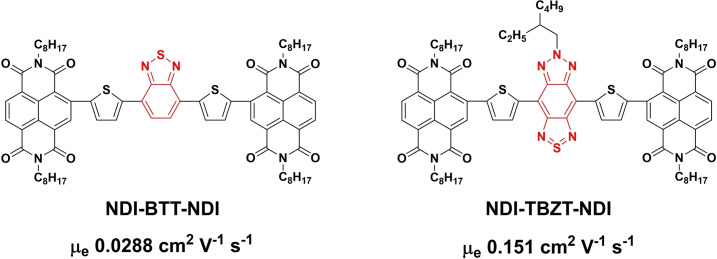

## 1 Introduction

Organic semiconducting materials have attracted significant attention due to their unique advantages such as solution‐processability, low‐cost and large‐area fabrication of thin film devices, and controlling the mechanical and flexible properties.^[1]^ Organic field‐effect transistors (OFETs), organic thin‐film solar cells (OSCs), organic light‐emitting diodes (OLEDs), and related electronic and photonic devices are the research targets using organic semiconductors.^[2]^ Among them, OFETs are attracting attention for their potential applications in flexible displays and logic circuits.^[3]^ The key properties of organic semiconductors required for high‐performance OFETs include frontier energy levels suitable for efficient charge injection, intermolecular overlap of molecular orbitals to enhance the carrier mobilities, and excellent operational stability under ambient conditions. As a result of molecular structural optimization, p‐type organic semiconductors have been reported to outperform amorphous silicon.^[4]^ However, the development of n‐type organic semiconductors has lagged behind that of p‐type semiconductors, although logic circuits and other electronic devices require both p‐type and n‐type semiconductors.^[5]^ The main problem is the intrinsic difficulty in forming air‐stable n‐channel OFETs due to the degradation of the electron‐transporting properties resulting from the reaction of radical anions with water in the air. One solution is to lower the LUMO levels of n‐type semiconductors as deep as −4.0 eV, which is expected to prevent the undesired side reactions with water.^[6]^ Rylene derivatives, such as naphthalenediimide (NDI) and perylenediimide (PDI), are commonly used building blocks of n‐type organic semiconductors because of their good electron affinity.^[7]^ In order to enhance the chemical stability, thiophene donors are often substituted with NDI and PDI. Moreover, it is well‐known that conjugation with the second acceptor unit enhances the electron‐transporting properties.^[8]^ For instance, in polymer semiconductors, donor‐acceptor_1_‐donor‐acceptor_2_ (D‐A_1_‐D‐A_2_) type backbone structures are an effective molecular design to form high mobility transistors. We previously reported the electron mobility (*μ*_e_) of 5.35 cm^2^ V^−1^ s^−1^ for the D‐A_1_‐D‐A_2_ type copolymer based on NDI and thiazolobenzotriazole (TBZ) acceptors (Figure S12).^[9]^ As a control, the D‐A_1_‐D‐A_2_ type copolymer based on NDI and benzothiadiazole (BT) acceptors displayed the *μ*_e_ of 0.92 cm^2^ V^−1^ s^−1^. It was suggested that the superior performance of the TBZ copolymer is mainly due to the deeper LUMO and better film morphology. However, some details are not clearly defined and require further investigation.

In this study, we decided to reveal the effects of the BT and TBZ acceptors on the electron‐transporting properties by designing model oligomers, namely, NDI‐BTT‐NDI and NDI‐TBZT‐NDI (BTT and TBZT refer to benzothiadiazolebisthiophene and thiazolobenzotriazolebisthiophene, respectively). Thin film transistors based on both NDI‐BTT‐NDI and NDI‐TBZT‐NDI were fabricated, and their performances were discussed in terms of energy levels, contact resistance, and film morphology.

## 2 Results and Discussion

### 2.1 Synthesis and Characterization

**Scheme 1:**
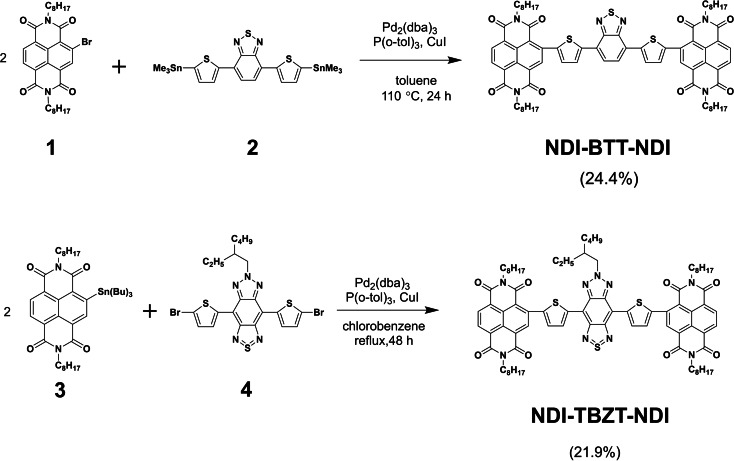
Synthesis of NDI‐BTT‐NDI and NDI‐TBZT‐NDI by Stille coupling reaction.

Scheme 1 shows the chemical structures and synthetic routes of NDI‐BTT‐NDI and NDI‐TBZT‐NDI. The CuI‐assisted Stille coupling between the brominated naphthalenediimide (NDI) derivative **1** and distannylated benzothiadiazoledithiophene (BTT) **2** afforded the desired NDI‐BTT‐NDI in 24.4% yield. This reaction also produced the mono‐NDI adduct, namely, NDI‐BTT, in 38.9% yield, suggesting the labile trimethyltin unit of **2** (Scheme S1). Due to the difficulty in preparing the stannylated thiazolobenzotriazoledithiophene (TBZT) derivatives, the functional groups were inverted in order to synthesize NDI‐TBZT‐NDI. In other words, the CuI‐assisted Stille coupling between the tributyltin‐substituted NDI **3** and dibrominated TBZT **4** was performed to produce the target NDI‐TBZT‐NDI in 21.9% yield. In this case, no mono‐adducts were isolated. The chemical structures of NDI‐BTT‐NDI and NDI‐TBZT‐NDI were characterized by their NMR, IR, and MALDI‐TOF‐MS spectra. NDI‐BTT‐NDI had a good solubility in the common organic solvents, such as chloroform, THF, and toluene. However, the solubility of NDI‐TBZT‐NDI in these solvents was limited, making it difficult to measure the ^13^C NMR spectrum.

The thermal properties were investigated by thermogravimetric analysis (TGA) and differential scanning calorimetry (DSC). The 5% weight loss temperatures (*T*_d5%_) of NDI‐BTT‐NDI and NDI‐TBZT‐NDI were 449 and 407 °C, respectively, suggesting the high thermal stability of these molecules (Figure S13). In the DSC thermograms, the first heating scan of NDI‐BTT‐NDI revealed two transition peaks at 212 and 220 °C (Figure S14). The second heating scan suggested that the peak at 212 °C is caused by the thermal history formed during the solid deposition, and accordingly, the melting temperature was determined to be 220 °C (Table 1). In contrast, NDI‐TBZT‐NDI showed a small exothermic peak at 217 °C in the first heating scan and this peak almost disappeared in the second heating. These results indicated that NDI‐TBZT‐NDI is more amorphous than NDI‐BTT‐NDI (*vide infra*).

**Table 1:** Summary of thermal, optical, and electrochemical properties.

	*T*_d 5%_ [°C]^a^	*T*_m_ [°C]^b^	*λ*_onset,film_ [nm]	*E*_g_^opt^ [eV]^c^	*E*_ox_ [V]	*E*_red_ [V]	*E* ^HOMO^ [eV]^d^	*E* ^LUMO^ [eV]^d^	*E*_g_^CV^ [eV]
NDI‐BTT‐NDI	449	220	743	1.67	1.65	−1.31	−6.45	−3.49	2.96
NDI‐TBZT‐NDI	407	217	918	1.35	1.47	−1.15	−6.27	−3.65	2.62

[a] Temperature at which a 5% weight loss occurred; [b] Melting point; [c] Optical bandgap estimated from the onset wavelength of the as‐spun films; [d] HOMO and LUMO energy levels measured for the thin films in CH_3_CN solution with 0.1 M (*n*C_4_H_9_)_4_NPF_6_ at the scan rate of 0.1 V s^−1^. For calibration, the redox potential of ferrocene/ferrocenium (Fc/Fc^+^) was measured under the same conditions.

### 2.2 Optical and Electrochemical Properties

**Figure 1:**
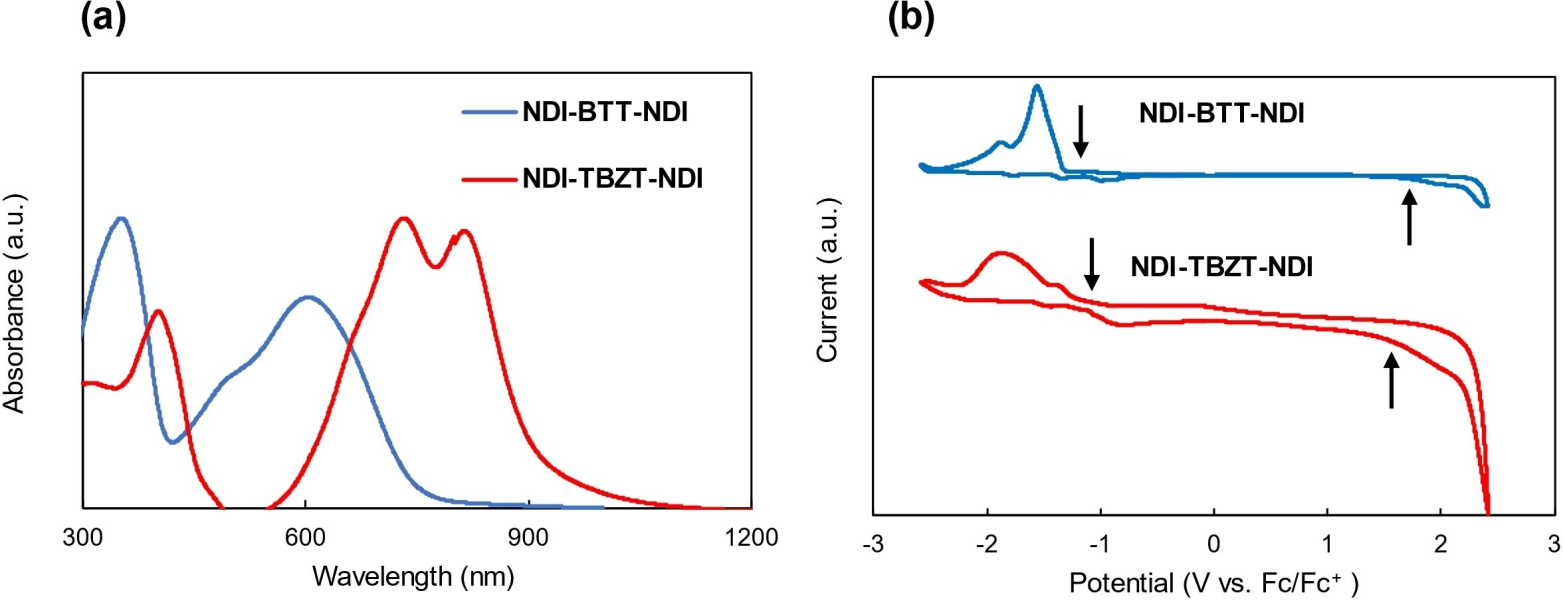
(a) Normalized UV‐vis‐near IR absorption spectra and (b) cyclic voltammograms (CVs) of the thin films of NDI‐BTT‐NDI (blue) and NDI‐TBZT‐NDI (red). The CVs were measured in CH_3_CN with 0.1 M (*n*C_4_H_9_)_4_NPF_6_. The first oxidation and reduction peaks are indicated by arrows.

In order to evaluate the optical properties and electronic energy levels, the UV‐vis‐near IR absorption spectra and cyclic voltammograms (CVs) of the solid films were measured. All the data are summarized in Table 1. For the absorption spectral measurements, the thin films of NDI‐BTT‐NDI and NDI‐TBZT‐NDI were prepared by drop‐casting CHCl_3_ solutions onto a glass substrate. NDI‐TBZT‐NDI showed a greater bathochromically shifted absorption with the *λ*_max_ of 814 nm than NDI‐BTT‐NDI with the *λ*_max_ of 604 nm, which is consistent with the chemical structure of the central acceptor unit (Figure 1a). TBZ has a more extended conjugation than BT. The optical energy bandgaps were estimated from the onset of the absorption wavelengths. NDI‐TBZT‐NDI had a narrower bandgap of 1.35 eV (918 nm) than NDI‐BTT‐NDI with the bandgap of 1.67 eV (743 nm). This result is also consistent with the previous polymer results of P(NDI‐BTT) and P(NDI‐TBZT) (for chemical structures, see Figure S12).^[9]^ Interestingly, the absorption spectra of NDI‐BTT‐NDI and NDI‐TBZT‐NDI in the THF solutions at the concentration of 10^−5^ M were blue‐shifted as compared to the thin film spectra and their spectral shapes were completely different (Figure S15). These results suggested that the longest wavelength peaks of the thin films originated from the strong intermolecular interactions.

The highest occupied molecular orbital (HOMO) and lowest unoccupied molecular orbital (LUMO) energy levels were estimated from the onset potentials of the first oxidation and first reduction of the thin films, respectively, measured in CH_3_CN with 0.1 M (*n*C_4_H_9_)_4_NPF_6_. Both NDI‐BTT‐NDI and NDI‐TBZT‐NDI displayed irreversible oxidation and reduction peaks (Figure 1b). The HOMO levels were as deep as −6.3∼−6.5 eV and the LUMO levels were −3.5∼−3.7 eV (Table 1). NDI‐TBZT‐NDI had a shallower HOMO and deeper LUMO than NDI‐BTT‐NDI due to the triply fused narrow bandgap unit of TBZ.

In order to visualize the distribution of the frontier molecular orbitals and three‐dimensional optimized structures, DFT calculations were performed using the B3LYP/6‐311+g method. In both NDI‐BTT‐NDI and NDI‐TBZT‐NDI, the HOMOs were localized on the central BTT and TBZT units, respectively (Figure S16). On the other hand, there was a well‐defined difference in the LUMOs. The LUMO of NDI‐BTT‐NDI was most likely localized on the NDI units, while NDI‐TBZT‐NDI had a significantly delocalized LUMO over the entire molecule. This result suggested the superior electron transporting property of NDI‐TBZT‐NDI.

### 2.3 Fabrication and Characterization of Organic Thin Film Transistors

**Figure 2:**
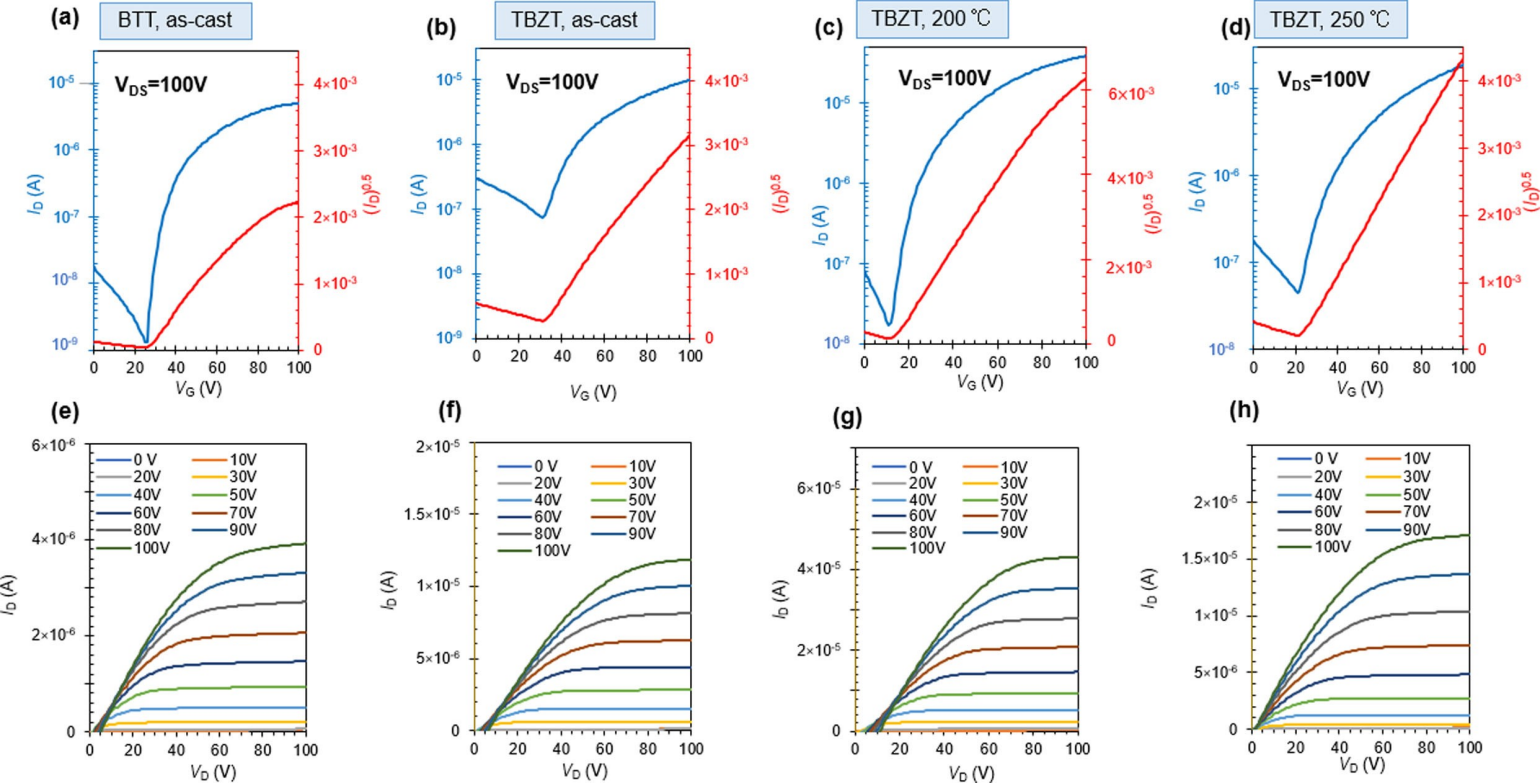
Organic field‐effect transistor performances of (a‐d) transfer characteristics and (e‐h) output characteristics based on NDI‐BTT‐NDI under n‐type operation (a,e: as‐cast) and NDI‐TBZT‐NDI under n‐type operation (b,f: as‐cast, c,g: after annealing at 200 °C for 10 min, d,h: after annealing at 250 °C for 10 min), measured in a vacuum (transistors have a channel length of *L*=100 μm and a width of *W*=1 mm).

In order to evaluate the charge transport properties, organic field‐effect transistor (OFET) devices with a bottom‐gate/top‐contact configuration were fabricated. The surface of the Si^++^/SiO_2_ substrates was initially modified by octadecyltrimethoxysilane (OTMS). After fabricating organic semiconducting layers by spin‐coating the chloroform solutions, they were subjected to a thermal annealing at selected temperatures (150, 200, or 250 °C) for 10 minutes. Lastly, ∼50 nm gold was deposited as the source and drain electrodes using a shadow mask. Typical transfer and output curves of the n‐channel OFETs are shown for both devices based on NDI‐BTT‐NDI and NDI‐TBZT‐NDI (Figure 2a–d). As NDI‐TBZT‐NDI displayed ambipolar characteristics, the p‐channel OFET performances are also shown in Figure S17. It should be noted that NDI‐BTT, which was obtained as a side product, did not exhibit any transistor performances despite the same structural units as NDI‐BTT‐NDI. In contrast, NDI‐BTT‐NDI exhibited unipolar n‐type characteristics. All the OFET performances are summarized in Table 2. The device based on NDI‐TBZT‐NDI showed much higher electron mobilities than those based on NDI‐BTT‐NDI, a result that is consistent with the previous report of the corresponding semiconducting polymers (for chemical structures, see Figure S12).^[9]^ The different OFET performances of NDI‐BTT‐NDI and NDI‐TBZT‐NDI are partly ascribed to the HOMO/LUMO energy levels. NDI‐TBZT‐NDI had a shallower HOMO and deeper LUMO than NDI‐BTT‐NDI, which led to the ambipolar behavior of NDI‐TBZT‐NDI. In addition to the energy levels, the film morphology affected the OFET results. Thermal annealing at the appropriate temperature improved the electron mobility of the NDI‐TBZT‐NDI‐based devices, and the device annealed at 200 °C exhibited the highest electron mobility (*μ*_e_=0.151 cm^2^ V^−1^ s^−1^). In contrast, for the NDI‐BTT‐NDI, thermal treatment at 200 and 250 °C caused the disappearance of the transistor characteristics. This was due to the formation of random crystalline aggregates (*vide infra*), which are not suitable for thin film transistor operation.

**Table 2:** Summary of OFET performances.

	Annealing temp. [°C]	*μ*_h_average_ [10^−3^ cm^2^ V^−1^ s^−1^]^a^	*μ*_h_max_ [10^−3^ cm^2^ V^−1^ s^−1^]	*μ*_e_average_ [10^−3^ cm^2^ V^−1^ s^−1^]^a^	*μ*_e_max_ [10^−3^ cm^2^ V^−1^ s^−1^]	*V*_th_ [V]	*I*_on_/*I*_off_
NDI‐BTT‐NDI	As‐cast	–	–	11.6±7.3	28.8	9.0±4.7	1.5×10^2^
	200	–	–	–	–	–	–
	250	–	–	–	–	–	–
NDI‐TBZT‐NDI	As‐cast	0.117±0.03	0.19	33.6±3.3	41.0	19.7±4.1	2.1×10^2^
	200	1.15±0.11	1.34	114±12	151	21.2±5.7	3.2×10^2^
	250	0.05±0.04	0.12	20.5±13.3	53.0	15.3±7.6	5.3×10^2^

[a] The average values were obtained from ∼12 devices in all cases.

**Figure 3:**
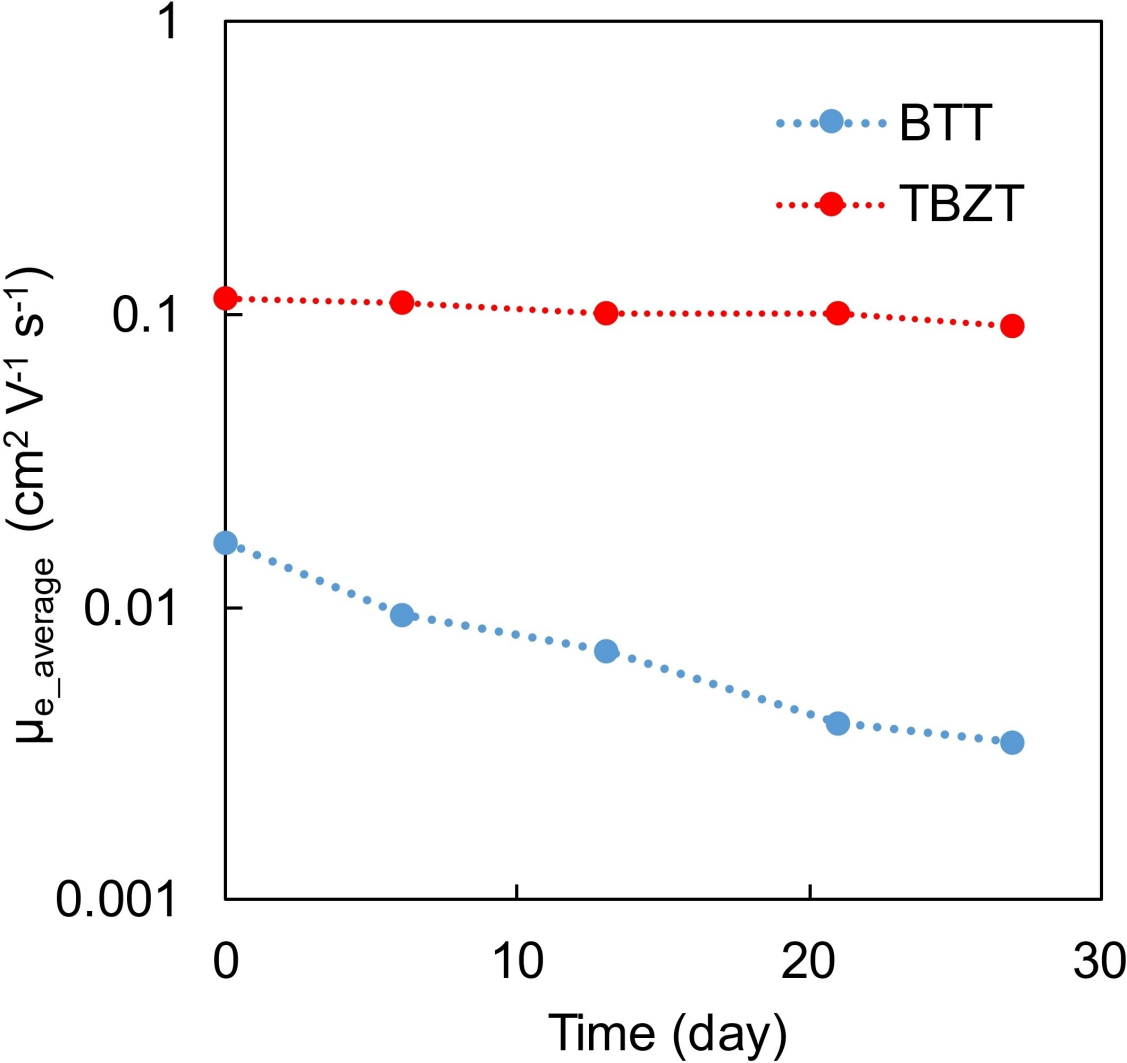
Time‐dependent change in the electron mobilities of OFETs based on NDI‐BTT‐NDI and NDI‐TBZT‐NDI stored in air.

In order to evaluate the air stability of the OFETs, the time‐dependent change in the electron mobilities was investigated. The best performing devices based on NDI‐BTT‐NDI and NDI‐TBZT‐NDI were fabricated. In other words, the NDI‐BTT‐NDI‐based device without any thermal treatment and the NDI‐TBZT‐NDI‐based device annealed at 200 °C were stored under ambient conditions and their transistor characteristics were then measured once a week. Although the electron mobility of NDI‐BTT‐NDI decreased to 20% of the initial value after one month, that of NDI‐TBZT‐NDI retained 80% under the same conditions (Figure 3). Note that the hole mobility of NDI‐TBZT‐NDI displayed a similar trend: No significant decrease in the *μ*_h_ was observed (Figure S18). One of the main factors in producing stable electron‐transporting properties is the LUMO level. It is commonly recognized that the LUMO levels as deep as −4.0 eV are required to achieve the air‐stable n‐channel operation of the OFETs.^[6]^ Although the LUMO level of NDI‐TBZT‐NDI estimated from the *E*_red_ value was −3.65 eV, it was apparently deeper than that of NDI‐BTT‐NDI (−3.49 eV). This result suggested that the TBZ unit is a promising key structure to produce air‐stable n‐type organic semiconductors.

**Figure 4:**
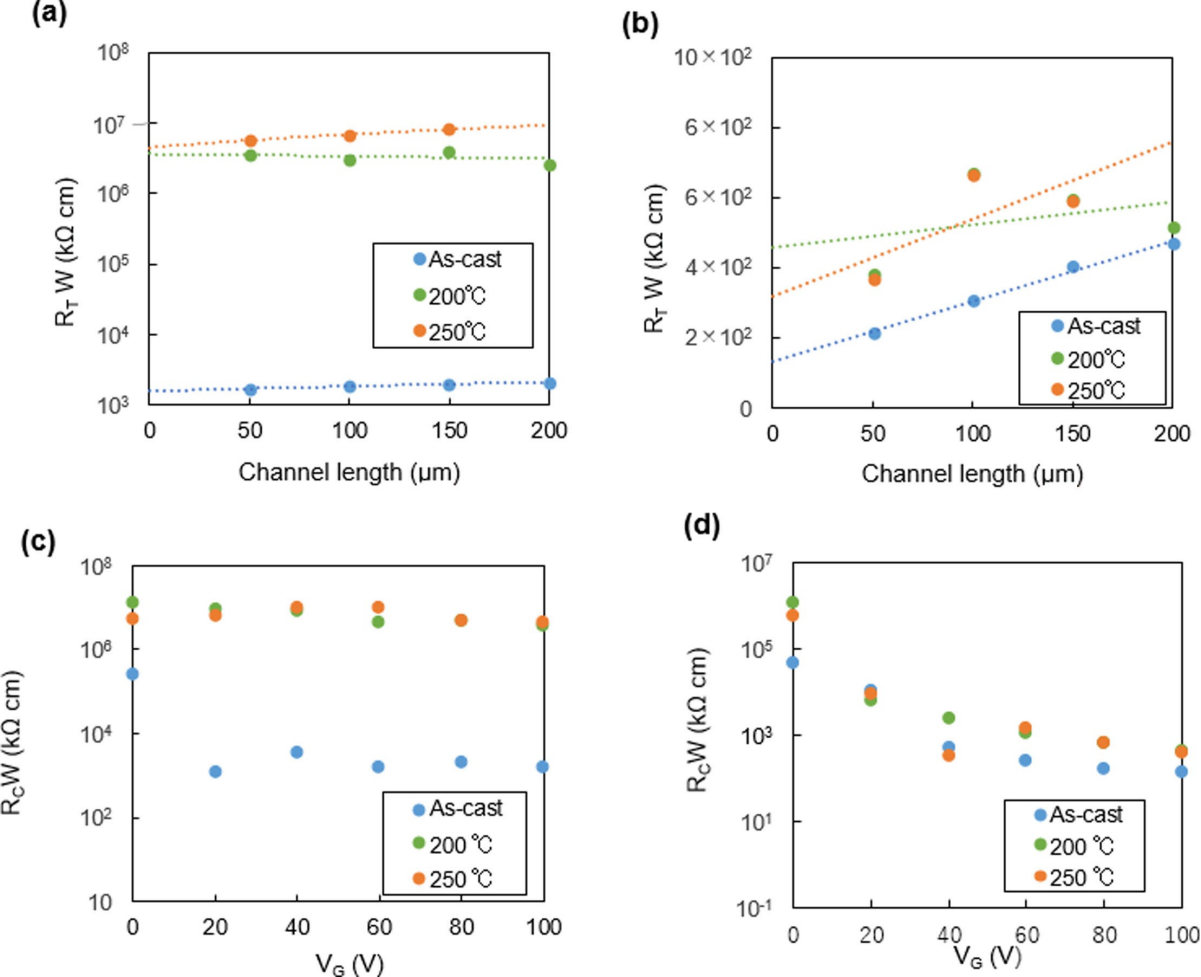
(a–b): Channel length dependence of width‐normalized total resistance (*R*_T_ *W*) with Au contact of OFETs based on (a) NDI‐BTT‐NDI and (b) NDI‐TBZT‐NDI under different annealing conditions (*V*_G_=100 V). (c–d): Gate voltage dependence of contact resistance (*R*_c_ *W*) of (c) NDI‐BTT‐NDI and (b) NDI‐TBZT‐NDI.

Moreover, to reveal another factor in determining the OFET performances, the effect of resistance at the metal‐organic interface was investigated. To this end, the contact resistance (*R*_c_) was extracted by a transfer line method.^[10]^ The contact resistance is a good parameter to understand the interfacial state between organic semiconductors and Au electrode.^[11]^ In general, a high contact resistance suggests a high barrier for charge injection. The device structure for the contact resistance measurements was the same as the top contact/bottom gate (TC/BG) configuration. The spin‐coated thin films of NDI‐BTT‐NDI and NDI‐TBZT‐NDI were prepared at different annealing temperatures, and the Au source and drain electrodes were deposited with several channel lengths, such as 50, 100, 150, and 200 μm on each device. The channel length dependence of the total resistance was measured by changing the gate voltages. The width‐normalized total resistance was plotted as a function of the channel lengths (Figure 4a–b), and the calculated contact resistance data are summarized in Table 3. It was shown that the contact resistance values have a clear correlation with the electron mobilities of the OFETs. In particular, the NDI‐BTT‐NDI‐based OFETs annealed at 200 and 250 °C, which did not show any transistor characteristics, had quite high contact resistances (∼10^6^ kΩ cm). This result indicated that the inferior contact between the thermally‐annealed NDI‐BTT‐NDI layer and Au electrode inhibited an efficient charge injection. However, the as‐cast film of NDI‐BTT‐NDI had a lower contact resistance of ∼10^3^ kΩ cm, and the films of NDI‐TBZT‐NDI showed a much lower contact resistance of ∼10^2^ kΩ cm. The contact resistance of these films was further decreased by applying higher gate voltages (Figure 4c–d and S19). All these results indicated that the resistance at the metal‐organic interface significantly affects the OFET performances.

**Table 3:** Contact resistance of OFETs based on NDI‐BTT‐NDI and NDI‐TBZT‐NDI.

	Annealing temp. [°C]	*R*_c_ *W* [kΩ cm]
NDI‐BTT‐NDI	As cast	1.59×10^3^
	200	3.64×10^6^
	250	5.79×10^6^
NDI‐TBZT‐NDI	As cast	1.33×10^2^
	200	4.59×10^2^
	250	4.13×10^2^

### 2.4 Film Morphology

In order to reveal the crystallinity, molecular orientations and surface morphology, the thin films were investigated in more detail. The thin film samples were prepared by the same method as the transistor devices on the Si^++^/SiO_2_ substrate modified with OTMS. To discuss the influence of thermal annealing on the film morphology, three different samples (as‐cast, annealed at 200 °C for 10 min, and annealed at 250 °C for 10 min) were prepared for each molecule.

**Figure 5:**
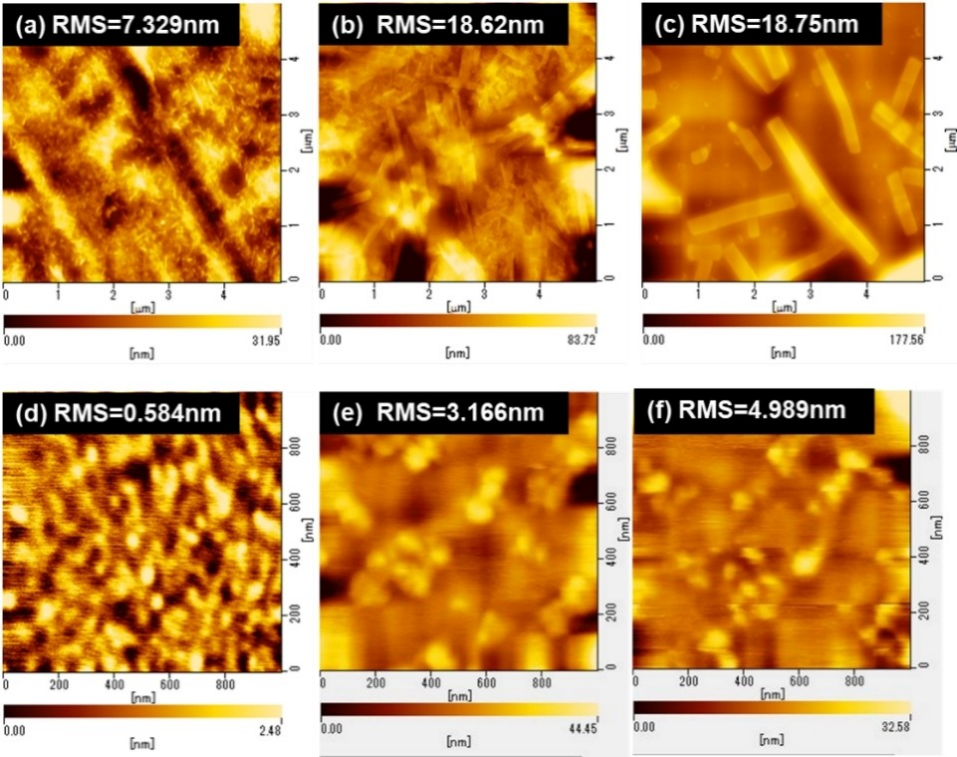
AFM images of (a‐c) NDI‐BTT‐NDI and (d‐f) NDI‐TBZT‐NDI thin films on OTMS‐treated Si^++^/SiO_2_ substrates. (a,d): as‐cast, (b,e): after annealing at 200 °C for 10 min, (c,f): after annealing at 250 °C for 10 min.

Tapping‐mode atomic force microscopy (AFM) was employed to investigate the surface morphologies (Figure 5). The as‐cast thin film of NDI‐BTT‐NDI showed an amorphous surface, while it displayed rod‐like structures upon annealing at 200 °C. The rod‐like structures became larger when the annealing temperature was increased to 250 °C. Accordingly, the root‐mean‐square (RMS) values of these annealed films were more than two times higher than that of the as‐cast film. Especially, large crystalline domains of several μm size were formed upon annealing at 250 °C. This may be due to the intrinsic high crystallinity of NDI‐BTT‐NDI, as suggested by the DSC curve (Figure S14). Generally, a high crystallinity of organic semiconductors leads to efficient charge‐carrier transport properties within the crystals. However, in this case, the formation of crystal domains deteriorated the smooth thin film morphology and operation of thin film transistors. On the other hand, the as‐cast film of NDI‐TBZT‐NDI showed uniform amorphous structures with a lower RMS of 0.584 nm (Figure 5d), which was consistent with the DSC curve (Figure S14). Thermal treatment enhanced the RMS value due to the formation of large crystalline grain sizes, but the thin film morphology was retained even after annealing at 250 °C. All these considerations well explained the observed transistor performances.

**Figure 6:**
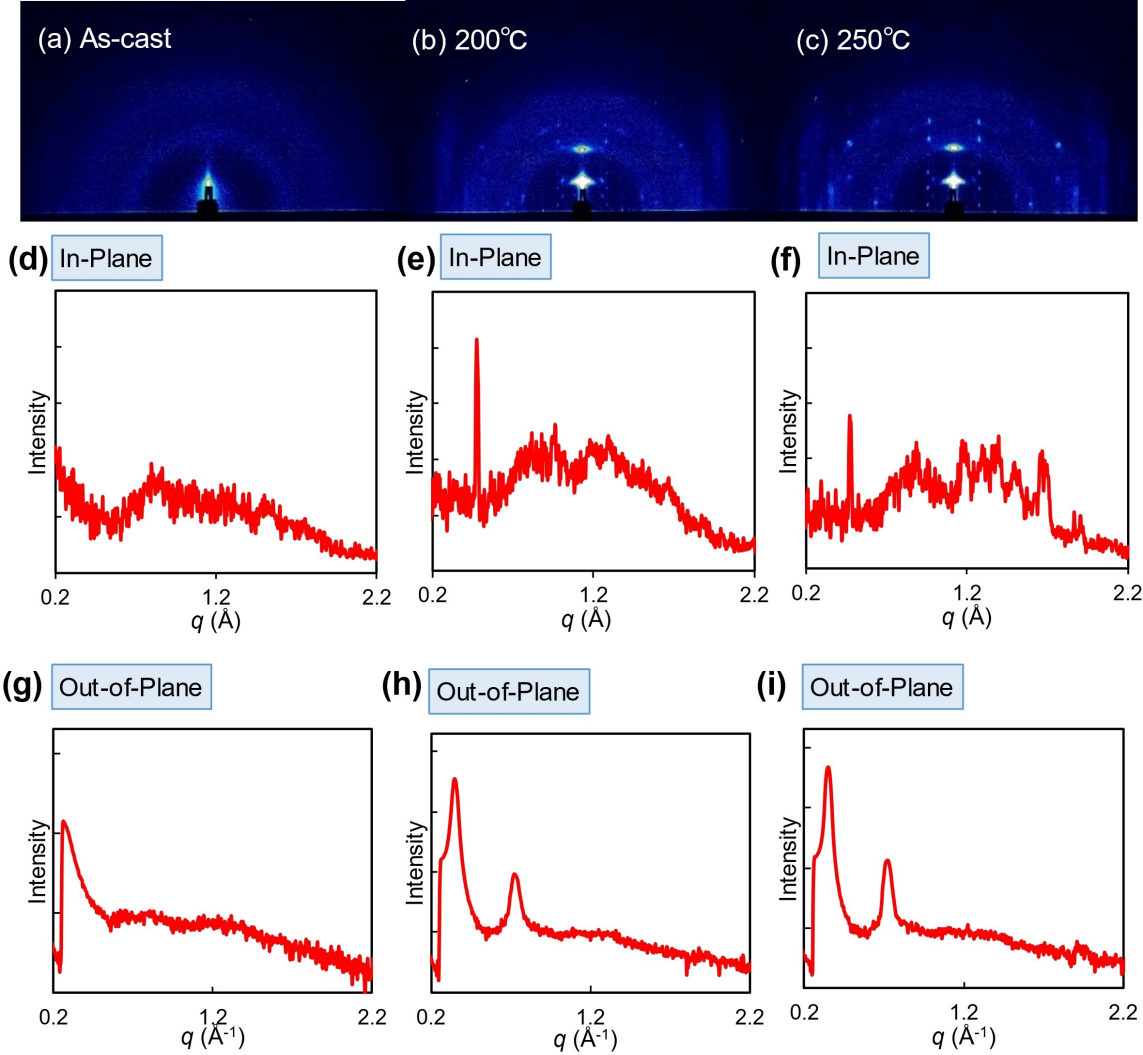
(a–c) 2D‐GIWAXS patterns of NDI‐BTT‐NDI thin films and (d–i) 1D profiles in the in‐plane and out‐of‐plane directions.

**Figure 7:**
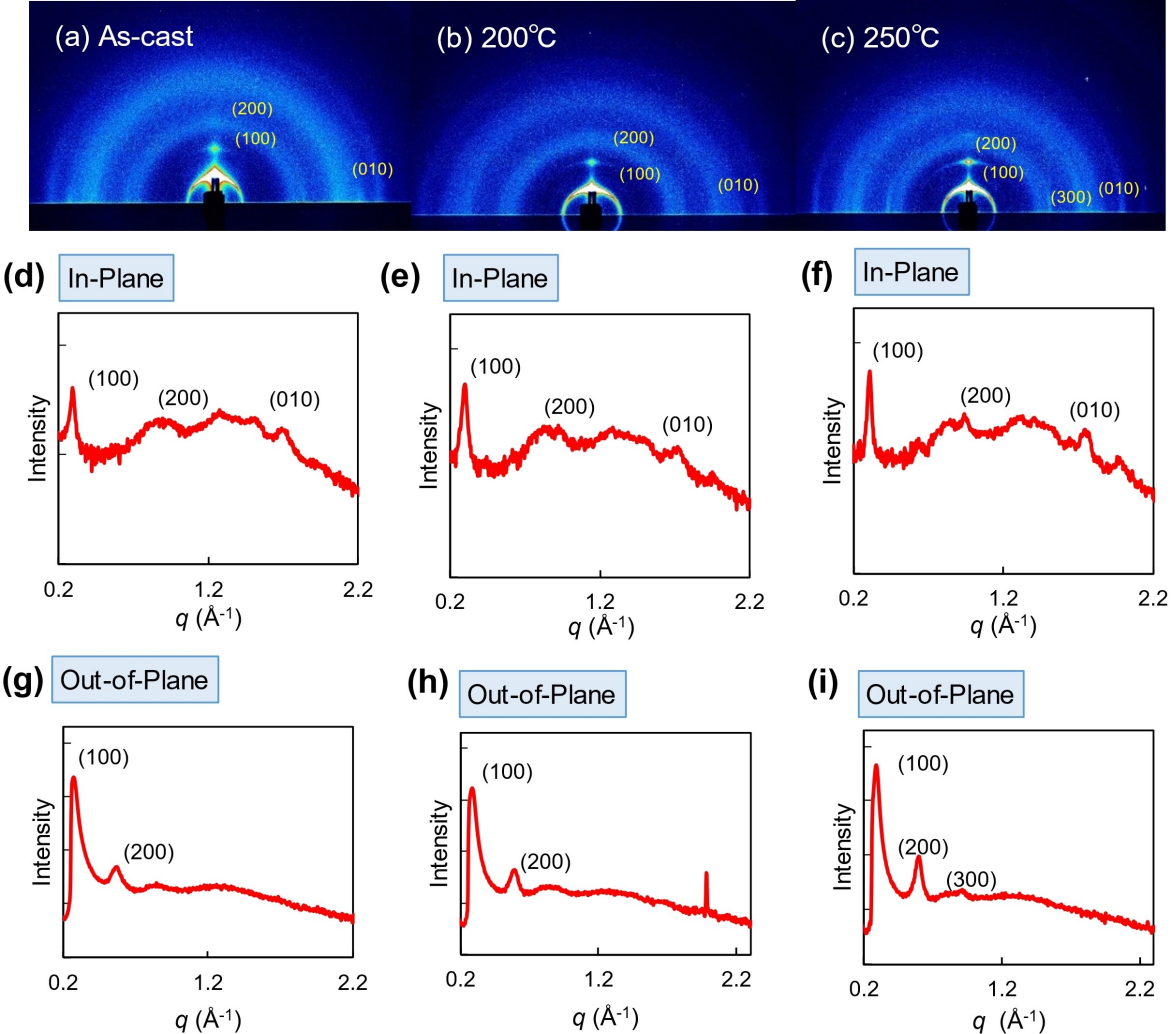
(a–c) 2D‐GIWAXS patterns of NDI‐TBZT‐NDI thin films and (d–i) 1D profiles in the in‐plane and out‐of‐plane directions.

In order to investigate the crystalline feature and molecular orientations in the thin films, 2D grazing‐incidence wide‐angle X‐ray scattering (2D‐GIWAXS) measurements were carried out. The 2D patterns and 1D diffraction profiles of NDI‐BTT‐NDI and NDI‐TBZT‐NDI are shown in Figure 6 and Figure 7, respectively. The molecular lengths and π‐π stacking distances, estimated from the observed peaks, as well as the coherence lengths are summarized in Table 4. Although the as‐cast film of NDI‐BTT‐NDI exhibited no well‐defined peaks, the annealed films showed sharp and intense diffractions at *q*=0.349 and 0.723° (*d*=18.0 and 8.7 Å) in the out‐of‐plane direction (Figure 6e,f) and 1.668° (*d*=3.8 Å) in the in‐plane direction (Figure 6h,i). These peaks suggested the high crystallinity and ordered packing of the annealed NDI‐BTT‐NDI thin films, which are consistent with the surface morphology observations by the AFM measurements. Moreover, the d‐spacing values did not directly correspond to the molecular length, indicating that the NDI‐BTT‐NDI molecules are aligned in a tilted configuration in the thin films. The annealed films at 200 and 250 °C did not significantly change the diffraction peak positions and coherent lengths.

**Table 4:** Summary of GIWAXS measurments of the NDI‐BTT‐NDI and NDI‐TBZT‐NDI thin films.

		Molecular length				π‐π stacking			
		*q* [Å^−1^]	*d* [Å]	FWHM [Å^−1^]	*L*_c_ [Å]	*q* [Å^−1^]	*d* [Å]	FWHM [Å^−1^]	*L*_c_ [Å]
NDI‐BTT‐NDI	NA	–	–	–	–	–	–	–	–
	200 °C	0.349	18.02	0.035	161.6	–	–	–	–
		0.723	8.692	0.038	148.8				
	250 °C	0.349	18.02	0.033	171.4	1.668	3.760	0.057	99.20
		0.723	8.692	0.046	122.9				
NDI‐TBZT‐NDI	NA	0.273	22.98	0.033	171.3	1.689	3.721	0.136	41.58
	200 °C	0.283	22.21	0.040	141.4	1.720	3.652	0.120	47.12
	250 °C	0.286	21.97	0.027	209.4	1.744	3.604	0.086	65.75

The thin films of NDI‐TBZT‐NDI displayed higher‐order diffraction peaks than the counter NDI‐BTT‐NDI. The d‐spacing values estimated from the in‐plane and out‐of‐plane directions were 21.7 and 22.9 Å, respectively. The d‐spacing was in good agreement with the computed molecular size with an all‐*trans* conformation for the alkyl chains. In addition, the (010) peak in the in‐plane direction suggested the edge‐on orientation of NDI‐TBZT‐NDI with the π‐π stacking distance of 3.72 Å. This value gradually decreased to 3.60 Å with the increasing annealing temperature indicating the enhanced crystallinity of the thin films. Moreover, in order to obtain information about the long‐range order, the crystalline coherence length (*L*_c_) was estimated. Noticeably, both the *L*_c_ (100) and *L*_c_ (010) values increased upon thermal annealing (Table 4). The best annealing temperature of 250 °C provided the *L*_c_ (100) of 209 Å and *L*_c_ (010) of 65.8 Å.

The thin films of both NDI‐BTT‐NDI and NDI‐TBZT‐NDI had many scattered peaks in the in‐plane and out‐of‐plane directions, suggesting the random orientation of the crystallites. In the case of polymer semiconductors, such structures are categorized into a bimodal orientation, suitable for high‐performance OFETs.^[12]^ In the NDI‐TBZT‐NDI devices, these structures may have partially contributed to the good transistor performances. In contrast, despite similar structures, the thermally‐annealed NDI‐BTT‐NDI films had no transistor characteristics because of the significantly high contact resistance at the interface between the semiconductor and electrode. Overall, in both cases of NDI‐BTT‐NDI and NDI‐TBZT‐NDI, the GIWAXS results were consistent with the surface morphology observed by AFM.

In order to get insight into the smooth contact between the semiconducting layer and Au electrode, the surface free energy of the thin films was investigated. Generally, the surface having a lower free energy is more hydrophobic and has a good contact with deposited electrodes. The contact angles of water and glycerol droplets dripped onto the thin films were measured (Figure S20) and the surface free energy was calculated from these angles.^[13]^ The measured contact angles and calculated surface free energies of each semiconductor film are summarized in Table S1. The thermal annealing treatment of the NDI‐BTT‐NDI films apparently enhanced the surface free energy from 14.48 to 15.27 mN m^−1^ (250 °C). On the contrary, the thermally‐annealed films of NDI‐TBZT‐NDI had lower surface free energies than those of the as‐cast films. In other words, the surface free energy of the as‐cast film was 16.52 mN m^−1^, but the value became 12.32 mN m^−1^ when annealed at 200 °C for 10 min. The values of the surface free energy thus had a good correlation with the OFET performances.

## 3 Conclusion

In reference to the previously reported high‐performance n‐type semoconducting polymers, NDI‐BTT‐NDI and NDI‐TBZT‐NDI were synthesized as the oligomeric models. It was found that the central acceptor units have a significant impact on the film morphology and charge‐transporting properties in the OFETs. NDI‐TBZT‐NDI showed the *μ*_e_ of 0.151 cm^2^ V^−1^ s^−1^ and high air‐stability with 80% of the initial mobility after one‐month storage under ambient conditions, while NDI‐BTT‐NDI displayed the *μ*_e_ of 0.0288 cm^2^ V^−1^ s^−1^ and limited air‐stability. The different OFET performances were investigated in detail. The deeper LUMO level and lower contact resistance of NDI‐TBZT‐NDI facilitated the electron injection as compared to NDI‐BTT‐NDI. In addition, uniform thin films with a moderate crystallinity were formed by spin‐coating the NDI‐TBZT‐NDI solution followed by a thermal annealing treatment. It was anticipated that all these positive features originate from the triply‐fused TBZ acceptor unit. Overall, it was shown that TBZ‐containing organic semiconductors are suitable for solution‐processable OFETs with air‐stable n‐channel performances.

## Experimental Section

### Materials

All chemicals were purchased from Tokyo Chemical Industry (TCI), Kanto Chemical Co. Inc, Sigma Aldrich, and Wako Pure Chemical Industries, and used as received without further purification.

### General Measurements

Nuclear magnetic resonance (NMR) spectra were recorded using a JEOL model AL300 (300 MHz) at room temperature. Deuterated chloroform was used as the solvent. Chemical shifts of NMR were reported in ppm (parts per million) relative to the residual solvent peak at 7.26 ppm for ^1^H NMR spectroscopy and 77.6 ppm for ^13^C NMR spectroscopy. Coupling constants (*J*) were given in Hz. The resonance multiplicity was described as s (singlet), d (doublet), t (triplet), and m (multiplet). Fourier transform infrared (FT‐IR) spectra were recorded by a JASCO FT/IR‐4200 spectrometer in the range from 4000 to 600 cm^−1^. MALDI‐TOF mass spectra were measured on a Shimadzu/Kratos AXIMACFR mass spectrometer equipped with a nitrogen laser (*λ*=337 nm) and pulsed ion extraction, which was operated in a linear‐positive ion mode at an accelerating potential of 20 kV. Tetrahydrofuran (THF) solutions containing 1 g L^−1^ of a sample, 20 g L^−1^ of dithranol, and 1 g L^−1^ of sodium trifluoroacetate were mixed to a ratio of 1 : 1 : 1; and then 1 μL aliquot of this mixture was deposited onto a sample target plate. UV‐vis‐near IR spectra were recorded on a JASCO V‐670 spectrophotometer. Thermogravimetric analysis (TGA) and differential scanning calorimetry (DSC) measurements were carried out on a Rigaku TG8120 and a Rigaku DSC8230, respectively, under nitrogen flow at the scan rate of 10 °C min^−1^. Contact angles were measured on a Phoenix 150/300 analyzer.

Electrochemistry measurements were carried out on a BAS electrochemical analyzer model 612 C at 20 °C in a classical three‐electrode cell. The working, reference, and auxiliary electrodes were a glassy carbon electrode, Ag/AgCl/CH_3_CN/(*n*C_4_H_9_)_4_NPF_6_, and a Pt wire, respectively. The solid films for electrochemical measurements were coated from a chloroform solution. For calibration, the redox potential of ferrocene/ferrocenium (Fc/Fc^+^) was measured under the same conditions. It was assumed that the redox potential of Fc/Fc^+^ has an absolute energy level of −4.80 eV to vacuum. The HOMO and LUMO energy levels were then calculated according to the following equations:

(1) $$E{}_{\mathrm{HOMO}}=-\left(\varphi {}_{\mathrm{ox}}+4.80\right)\;\left(\mathrm{eV}\right)$$

(2) $$E{}_{\mathrm{LUMO}}=-\left(\varphi {}_{\mathrm{re}}+4.80\right)\;\left(\mathrm{eV}\right)$$

where ϕ_ox_ is the onset oxidation potential vs Fc/Fc^+^ and ϕ_re_ is the onset reduction potential vs Fc/Fc^+^.

### OFET Device Fabrication and Characterization

Top‐contact/bottom‐gate (TC/BG) OFET devices were fabricated on n^++^‐Si substrates with 300 nm SiO_2_ where n^++^‐Si and SiO_2_ were used as the gate electrode and gate dielectric, respectively. The substrates were subjected to cleaning and modified with octadecyltrimethylsilane (OTMS) to form a self‐assembled monolayer (SAM). Thin films of the organic semiconducting molecules were deposited on the treated substrate by spin‐coating the solutions (5 mg/mL in chloroform; for NDI‐BTT‐NDI, 3000 rpm for 30 s and for NDI‐TBZT‐NDI, 1000 rpm for 30 s) inside an argon‐filled glovebox followed by optional thermal annealing at 150 °C, 200 °C, or 250 °C for 10 min. After the thin film deposition, ∼50 nm thick gold was deposited as source and drain contacts using a shadow mask. The OFET devices had a channel length (*L*) of 100 μm and a channel width (*W*) of 1 mm. The OFET performances were measured under dynamic vacuum (10^−5^ mbar) using a Keithley 4200A‐SCS parameter analyzer on a probe stage. The carrier mobilities, *μ*, were calculated from the data in the saturated regime according to the following equation:

(3) $$I{}_{\mathrm{SD}}=\left(W/2\;L\right)\;C{}_{i}\;\mu \;\left(V{}_{G}-V{}_{T}\right){}^{2}$$

where *I*_SD_ is the drain current in the saturated regime, *W* and *L* are the semiconductor channel width and length, respectively, *C*_i_ (*C*_i_=11.2 nF cm^−2^) is the capacitance per unit area of the gate dielectric layer, and *V*_G_ and *V*_T_ are the gate voltage and threshold voltage, respectively. *V*_G_−*V*_T_ of the devices was determined from the square root values of *I*_SD_ at the saturated regime. For n‐type characterization, *I*_on_/*I*_off_ values were determined from the minimum current at around *V*_GS_=40 V (*I*_off_) and the current at *V*_GS_=100 V (*I*_on_). The sweep direction in the transfer characteristics was from *V*_GS_=0 to 100 V. For p‐type characterization, *I*_on_/*I*_off_ values were determined from the minimum current at around *V*_GS_=−40 V (*I*_off_) and the current at *V*_GS_=−100 V (*I*_on_). The sweep direction in the transfer characteristics was from *V*_GS_=0 to −100 V.

### Atomic Force Microscopy (AFM) Measurements

AFM samples were prepared by spin‐coating the organic semiconductor solutions in chloroform onto an OTMS‐treated Si^++^/SiO_2_ substrate. Both pristine and thermally‐treated films were examined by a Seiko Instruments SPA‐400 with a stiff cantilever of Seiko Instruments DF‐20.

### Grazing‐Incidence Wide‐Angle X‐Ray Scattering (GIWAXS) Measurements

Grazing‐incidence wide‐angle X‐ray scattering (GIWAXS) measurements were carried out at BL40B2 in SPring‐8 (Hyogo, Japan). The wavelength of the X‐ray beam was 0.1 nm, and camera length was 343.6219 mm. The 2D‐scattering image was acquired using a photon counting detector (PILATUS3 S 2M, Dectris Ltd.). GIWAXS data were measured at the incident angle of 0.10°, which was lower than the critical angle of total external reflection at silicon surface and was close to those of samples. The components of the scattering vector, q, parallel and perpendicular to the sample surface were defined as q_xy_ and q_z_, respectively. Thin film samples for GIWAXS measurements were prepared by spin‐coating of the organic semiconductor solutions onto an OTMS‐treated Si^++^/SiO_2_ substrate.

### Contact Angle Measurements and Surface Energy Calculation

The samples for contact angle measurements were prepared by the same method as the transistor fabrication. Static contact angles of the thin films were measured by the sessile drop technique using deionized water and glycerol as probe liquids with a Surface Electro Optics Phoenix 150/300 Contact Angle Analyzer. The surface energies were calculated based on the Owens‐Wendt method.^[13]^

(4) $$\gamma_{water}\left(1+cos\theta_{water}\right)=\frac{4\gamma_{water}^{d}\gamma^{d}}{\gamma_{water}^{d}+\gamma^{d}}+\frac{4\gamma_{water}^{p}\;\gamma^{p}}{\gamma_{water}^{p}+\gamma^{p}}$$

(5) $$\gamma_{glycerol}\left(1+cos\theta_{glycerol}\right)=\frac{4\gamma_{glycerol}^{d}\gamma^{d}}{\gamma_{glycerol}^{d}+\gamma^{d}}+\frac{4\gamma_{glycerol}^{p}\;\gamma^{p}}{\gamma_{glycerol}^{p}+\gamma^{p}}$$

(6) $$\gamma^{total}=\gamma^{d}+\gamma^{p}$$

where *γ* ^total^ is the total surface free energy, *γ* ^d^ and *γ* ^p^ are the dispersion and polar components of surface free energy, respectively. The *γ*_water_ and *γ*_glycerol_ are the total free energy of water and glycerol, respectively, and *γ* ^d^_water_, *γ* ^p^_water_, *γ* ^d^_glycerol_ and *γ* ^p^_glycerol_ are their dispersion or polar components. *θ*_water_ and *θ*_glycerol_ are measured contact angles of water and glycerol droplets on films, respectively.

### Synthesis

NDI‐BTT‐NDI and NDI‐BTT: **1** (210.0 mg, 0.6050 mmol), **2** (181.1 mg, 0.2900 mmol), Pd_2_(dba)_3_ (86.0 mg, 0.0940 mmol), P(*o*‐tolyl)_3_ (13.5 mg, 0.0440 mmol), and CuI (10.7 mg, 0.0560 mmol) were dissolved into toluene (10 mL). The solution was stirred at 110 °C for 24 h under argon atmosphere. After cooling to room temperature, water was added and the organic phase was extracted with CH_2_Cl_2_ 3 times. The combined organic layer was dried over MgSO_4_ and concentrated. After column chromatography (SiO_2_, hexane/CH_2_Cl_2_=1 : 5) and recycling HPLC (chloroform), NDI‐BTT‐NDI (90.2 mg, 0.0705 mmol, 24.4%) and NDI‐BTT (88.2 mg, 0.0690 mmol, 24.0%) were obtained as the dark purple solid and dark red solid, respectively.

NDI‐BTT‐NDI: ^1^H NMR (CDCl_3_, 399.3 MHz, 293 K) δ [ppm] 8.81 (d, *J*=7.2 Hz, 1H), 8.80 (s, 2H), 8.74 (d, *J*=8.0 Hz, 1H), 8.20 (d, *J*=3.6 Hz, 2H), 7.96 (s, 2H), 7.44 (d, *J*=5.9 Hz, 2H), 4.21–4.13 (m, 8H), 1.78–1.68 (m, 8H), 1.47–1.22 (m, 40H), 0.94–0.83 (m, 12H); ^13^C NMR (CDCl_3_, 100.4 MHz, 293 K) δ [ppm] 162.74, 162.45, 162.09, 152.36, 142.48, 141.90, 140.22, 136.09, 131.45, 130.64, 129.61, 127.95, 127.67, 126.78, 126.52, 126.19, 125.80, 125.57, 125.21, 123.32, 41.26, 41.02, 31.81, 31.78, 29.32, 29.29, 29.23, 29.20, 28.07, 27.12, 27.08, 22.64, 22.62, 14.10; IR ν [cm^−1^] 2924.5, 2854.1, 1706.7, 1662.0, 1566.9, 1461.8, 1436.7, 1409.7, 1371.1, 1335.5, 1247.7, 1183.1, 786.8, 651.8, 633.5, 614.2, 605.5; MALDI‐TOF MS (dithranol); m/z calcd for C_74_H_81_N_6_O_8_S_3_^+^: 1277.53; found: 1276.80 [M+H]^+^; m/z calcd for C_74_H_80_N_6_NaO_8_S_3_^+^: 1299.51; found: 1299.81 [M+Na]^+^.

NDI‐BTT: ^1^H NMR (CDCl_3_, 399.3 MHz, 293 K) δ [ppm] 8.83–8.81 (m, 2H), 8.76–8.73 (m, 1H), 8.17–8.15 (m, 2H), 7.99–7.96 (m, 1H), 7.93–7.90 (m, 1H), 7.49 (d, *J*=6.0 Hz, 1H), 7.42 (d, *J*=3.2 Hz, 1H), 7.23 (t, *J*=3.6 Hz, 1H), 4.20 (t, *J*=6.8 Hz, 2H), 4.12 (t, *J*=11.2 Hz, 2H), 1.60 (m, 4H), 1.46–1.26 (m, 20H), 0.89–0.82 (m, 6H); ^13^C NMR (CDCl_3_, 100.4 MHz, 293 K) δ [ppm] 162.60, 162.32, 161.98, 152.23, 152.13, 142.18, 141.91, 140.05, 139.03, 135.91, 131.19, 130.29, 129.69, 128.06,127.33, 127.11, 126.50, 126.28, 126.16, 125.90. 125.44, 125.35, 125.07, 123.01, 41.16, 40.96, 31.80, 29.21, 28.06, 27.10, 22.62, 14.11; IR ν [cm^−1^] 3360.4, 2925.5, 2855.1, 1732.7, 1704.8, 1664.3, 1636.3, 1540.9, 1521.6, 1507.1, 1497.5, 1488.8, 1457.0, 1435.7, 1396.2, 1375.0, 1337.4, 1311.4, 1254.5, 1233.3, 1184.1, 1129.1, 1050.1, 876.5, 832.1, 786.8, 748.2, 719.3, 698.1, 686.5, 670.1, 641.2, 613.2; MALDI‐TOF MS (dithranol); m/z calcd for C_44_H_44_N_4_O_4_S_3_^+^: 788.25; found: 788.00 [M]^+^.

NDI‐TBZT‐NDI: **3** (186.0 mg, 0.2380 mmol), **4** (427.3 mg, 0.6930 mmol), Pd_2_(dba)_3_ (85.3 mg, 0.0930 mmol), P(*o*‐tolyl)_3_ (11.3 mg, 0.0370 mmol), and CuI (8.0 mg, 0.042 mmol) were dissolved into chlorobenzene (15 mL). The solution was stirred at 140 °C for 48 h under argon atmosphere. After cooling to room temperature, water was added and the organic layer was extracted with CH_2_Cl_2_ 3 times. The combined organic layer was dried over MgSO_4_ and concentrated. After column chromatography (SiO_2_, hexane/CH_2_Cl_2_=1 : 5) and recycling HPLC (chloroform), the targe compound was obtained as a dark green solid (74.7 mg, 0.0520 mmol, 21.9%).

^1^H NMR (CDCl_3_, 399.3 MHz, 293 K) δ [ppm] 9.00 (d, *J*=4.0 Hz, 2H), 8.86 (s, 2H), 8.78 (d, *J*=7.6 Hz, 2H), 8.67 (d, *J*=7.6 Hz, 2H), 7.55 (d, *J*=5.2 Hz, 2H), 4.94 (d, *J*=7.2 Hz, 2H), 4.21–4.13 (m, 8H), 2.45–2.40 (m, 1H), 1.77 (m, 6H), 1.46–1.26 (m, 50H), 1.06 (t, *J*=8.0 Hz, 2H), 0.94–0.82 (m, 16H); ^13^C NMR could not be measured due to the low solubility; IR ν [cm^−1^] 2956.3, 2923.6, 2854.1, 1699.9, 1653.7, 1615.1, 1657.8, 1540.9, 1517.7, 1488.8, 1458.9, 1436.7, 1408.8, 1376.0, 1339.3, 1284.4, 1265.1, 1182.2, 1098.3, 1049.1, 967.1, 932.4, 839.8, 809.0, 717.4, 677.9, 667.3, 650.9, 621.9, 608.4, 578.5, 565.0, 549.6, 521.7, 510.1; MALDI‐TOF MS (dithranol); m/z calcd for C_82_H_95_N_9_O_8_S_3_^+^: 1430.65; found: 1430.12 [M+H]^+^; m/z calcd for C_82_H_95_N_9_NaO_8_S_3_^+^: 1452.64; found: 1452.99 [M+Na]^+^.

## Conflict of interest

The authors declare no conflict of interest.

4

## Supporting information

As a service to our authors and readers, this journal provides supporting information supplied by the authors. Such materials are peer reviewed and may be re‐organized for online delivery, but are not copy‐edited or typeset. Technical support issues arising from supporting information (other than missing files) should be addressed to the authors.

Supporting Information

## Data Availability

The data that support the findings of this study are available from the corresponding author upon reasonable request.
