# Supplementary material for: Benzothiadiazole versus Thiazolobenzotriazole: A Structural Study of Electron Acceptors in Solution‐Processable Organic Semiconductors
Source: Chem Asian J. 2022 Sep 30;17(22):e202200768. doi: 10.1002/asia.202200768 (PMC9828094; doi:10.1002/asia.202200768)
Supplement: Supplementary file 1 — Supporting Information [file ASIA-17-0-s001.pdf]

CHEMISTRY
AN ASIAN JOURNAL

Supporting Information

Benzothiadiazole versus Thiazolobenzotriazole: A Structural Study of Electron Acceptors in Solution-Processable Organic Semiconductors

Nanami Watanabe, Waner He, Naoya Nozaki, Hidetoshi Matsumoto, and Tsuyoshi Michinobu*© 2022 The Authors. Chemistry - An Asian Journal published by Wiley-VCH GmbH. This is an open access article under the terms of the Creative Commons Attribution License, which permits use, distribution and reproduction in any medium, provided the original work is properly cited.

1. Synthesis

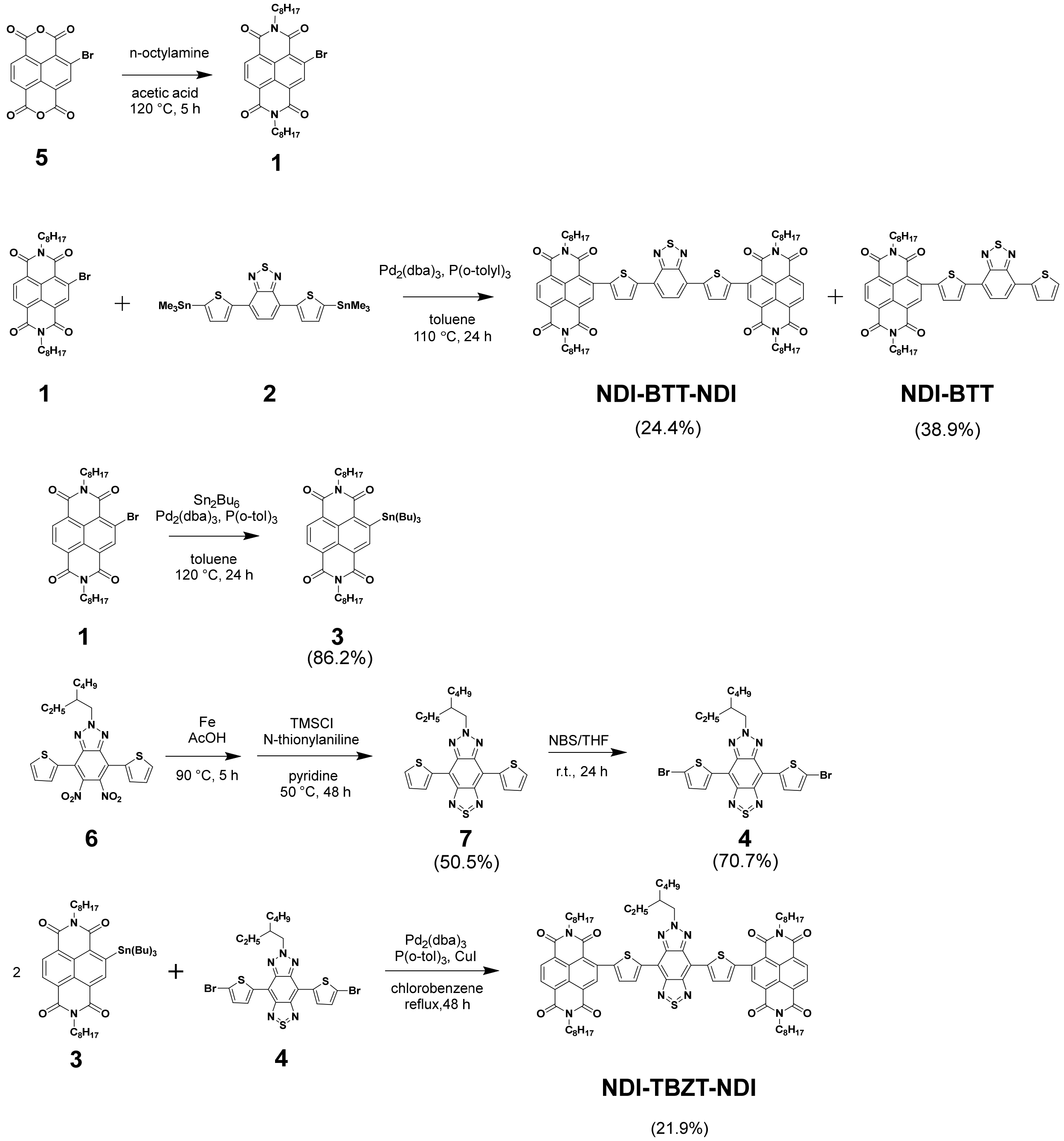

Scheme S1 Synthesis of NDI-BTT -NDI and NDI-TBZT-NDI.

1.1.Synthesis of 4-bromo-2,7-dioctylbenzo[*lmn*][3,8]phenanthroline-1,3,6,8(2*H*,7*H*)-tetrone (**1**)

2-Bromonaphthalene-1,4,5,8-tetracarboxylic 1,8:4,5-dianhydride (**5**) (499.7 mg, 1.44 mmol) and *n*-octylamine (5 mL) were dissolved into acetic acid (125 mL). The

solution was stirred for 5 h at 120 °C under the argon atmosphere. After cooling at room temperature, the resulting solution was poured into water (100 mL). The pale-orange participate was collected and washed with methanol. After column chromatography (SiO_2, hexane/CH_2Cl_2 =3:7), the target compound was obtained as the pale yellow solid (276 mg, 0.485 mmol, 33.7 %).

^{1}H NMR ($CDCl_3$, 399.3 MHz, 293 K) δ [ppm] 0.86-0.90 (m, 6H), 1.27-1.41 (m, 20H), 1.69-1.78 (m, 4H), 4.16-4.21 (m, 4H), 8.76-8.83 (m, 2H), 8.93 (s,1H).

1.2.Synthesis of 2,7-dioctyl-4-(tributylstannyl)benzo[*lmn*][3,8]phenanthroline-1,3,6,8(2*H*,7*H*)-tetraone (**3**)[S1]

A mixture of **1** (406.0 mg, 0.712 mmol), bis(tributyltin) (1.52 g, 1.86 mmol), $Pd_2(dba)_3$ (43.2 mg, 0.0380 mmol), and P(*o*-tolyl)$_3$ (145 mg, 0.476 mol) was dissolved in toluene (26 mL). The reaction mixture was stirred at 120 °C under argon atmosphere for 24 h. After cooling to room temperature, the resulting solution was evaporated and the mixture was purified by column chromatography (hexane/CH_2Cl_2 = 1:1) and recycling HPLC (chloroform), yielding the target compound as the yellow solid (479.1 mg, 0.614 mmol, 86.2 %).

^{1}H NMR ($CDCl_3$, 399.3 MHz, 293 K) δ [ppm] 0.86-0.91 (m, 15H), 1.25-1.53 (m, 38H), 1.72-1.74 (m, 4H), 4.16-4.23 (m, 4H), 8.70-8.75 (m, 2H), 8.97 (s, 1H).

1.3.Synthesis of 4,8-bis(thiophen-2-yl)-6-(2-ethylhexyl)[1,2,5]thiadiazolo[3,4-*f*]benzotriazole (**7**)[S2]

A 200 mL three-necked flask was charged with 2-(2-ethylhexyl)-5,6-dinitro-4,7-bis (thiophen- 2-yl)-2*H*-benzo[d] [1,2,3] triazole (**6**) (571.4 mg, 1.008 mmol), iron dust 1.266 mg, 0.02260 mmol), and acetic acid (37.5 mL). After the mixture was heated to 60 °C for 5 h, it was cooled to room temperature, diluted with water (75 mL), and extracted with CH_2Cl_2 (3 × 75 mL). The combined organic layers were washed with aq. $NaHCO_3$ solution (2 × 75 mL) and dried over $MgSO_4$. Filtration followed by solvent removal under reduced pressure yielded (2-(2- ethylhexyl)-4,7-bis(thiophen-2-

yl)-2*H*-benzo[*d*][1,2,3]triazole-5,6-diamine) as yellowish- brown oil that was relatively pure and directly used for the next step. The yellowish-brown oil was homogeneously mixed with dry pyridine (5.25 mL) under argon. To this solution, *N*-thionylaniline (0.255 mL, 2.25 mmol) followed by trimethylsilyl chloride (0.57 mL, 4.5 mmol) were added. After the mixture was stirred at 50 °C for 36 h, toluene (10 mL) was added and the solution was filtered through a silica gel plug. Evaporation gave a dark blue powder. Column chromatography (SiO_2, hexane/CH_2Cl_2 = 3:1) afforded the target compound as the dark bluish purple solid (258 g, 0.568 mmol, two-step yield of 50.5 %).

^{1}H NMR ($CDCl_3$, 399.3 MHz, 293 K) δ [ppm] 8.87(d, J = 3.6 Hz, 2H), 7.63 (d, J = 4.4 Hz, 2H), 7.31-7.33 (m, 2H), 4.90 (d, J = 6.8 Hz, 2H), 2.38-2.41 (m, 1H), 1.26-1.48 (m, 8H), 0.90 (t, J = 7.6Hz, 3H), 1.04 (t, J = 7.6 Hz, 3H)

1.4.Synthesis of 4,8-bis(5-bromothiophen-2-yl)-6-(2-ethylhexyl)[1,2,5]thiadiazolo[3,4-*f*]benzotriazole (**4**)[S2]

4,8-Bis(thiophen-2-yl)-6-(2-ethylhexyl)[1,2,5]thiadiazolo[3,4-*f*]benzotriazole (**7**) (200.4 mg, 0.4410 mmol) was dissolved in dry THF (20 mL) under N_2 atmosphere. *N*-Bromosuccinimide (164.4 mg, 0.9560 mmol) was added several portions over the course of 10 min at room temperature in the dark. After the mixture was stirred for 2 h and poured into water (75 mL), it was extracted with CH_2Cl_2 (75 mL). After evaporation, the crude product was purified by column chromatography (SiO_2, hexane/CH_2Cl_2 = 2:1) to afford the target compound as the dark blue solid (243.8 mg, 71.0 %).

^{1}H NMR ($CDCl_3$, 399.3 MHz, 293 K) δ [ppm] 8.38 (d, J = 4.0 Hz, 2H), 7.16 (d, J = 4.0 Hz, 2H), 4.75 (d, J = 6.8 Hz, 2H), 2.24-2.32 (m, 1H), 1.24-1.43 (m, 8H), 0.90-1.03 (m, 6H).

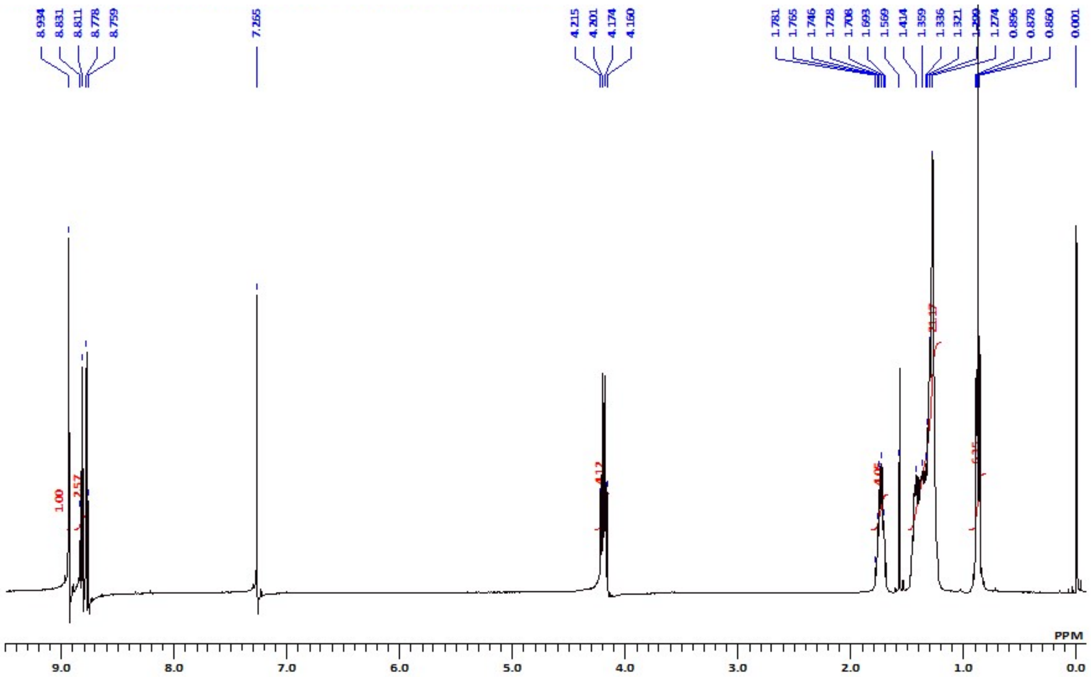

Figure S1 ^{1}H NMR spectrum of **1** in $CDCl_3$.

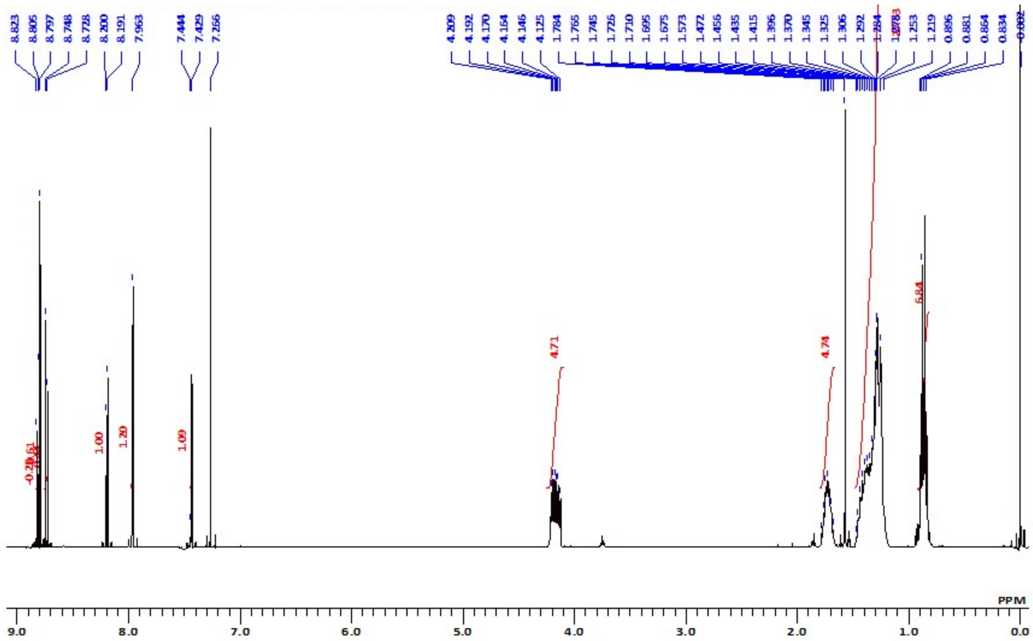

Figure S2 ^{1}H NMR spectrum of NDI-BTT-NDI in $CDCl_3$.

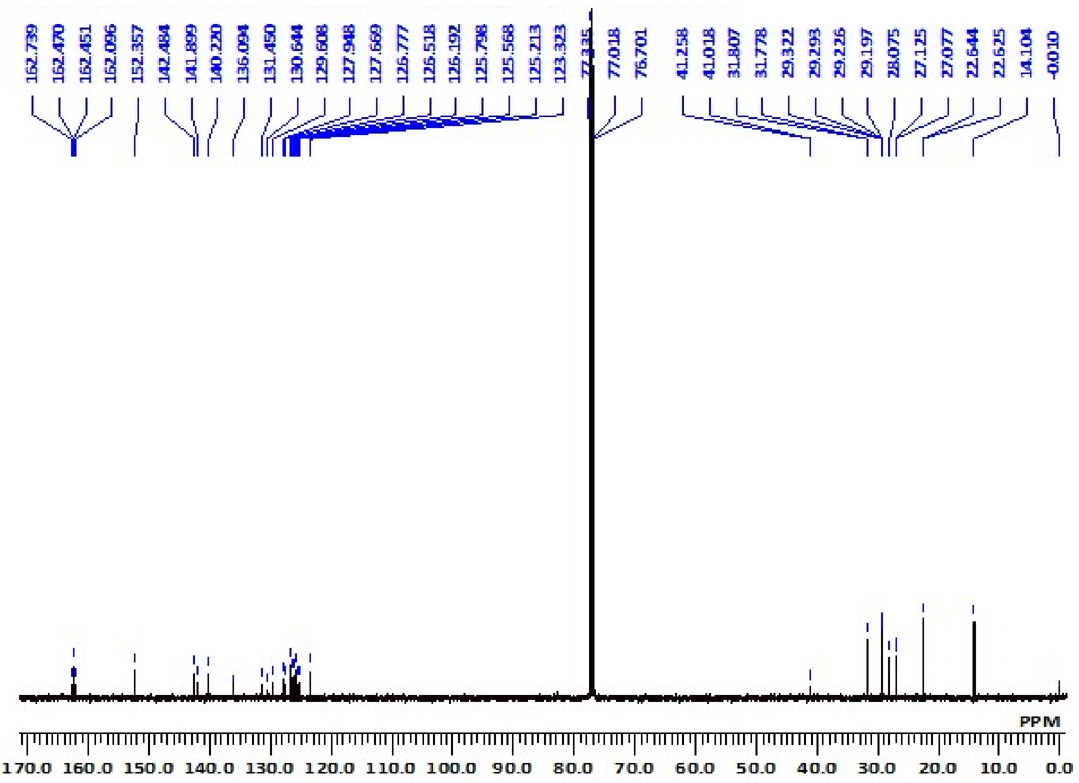

Figure S3 ^{13}C NMR spectrum of NDI-BTT-NDI in $CDCl_3$.

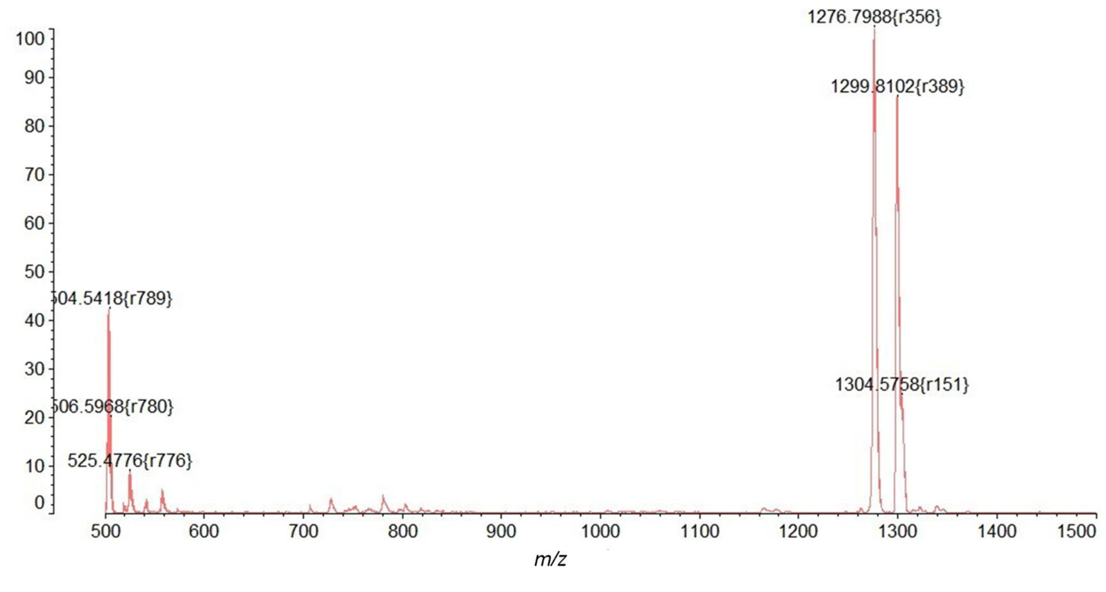

Figure S4 MALDI-TOF mass spectrum of NDI-BTT-NDI (matrix: dithranol).

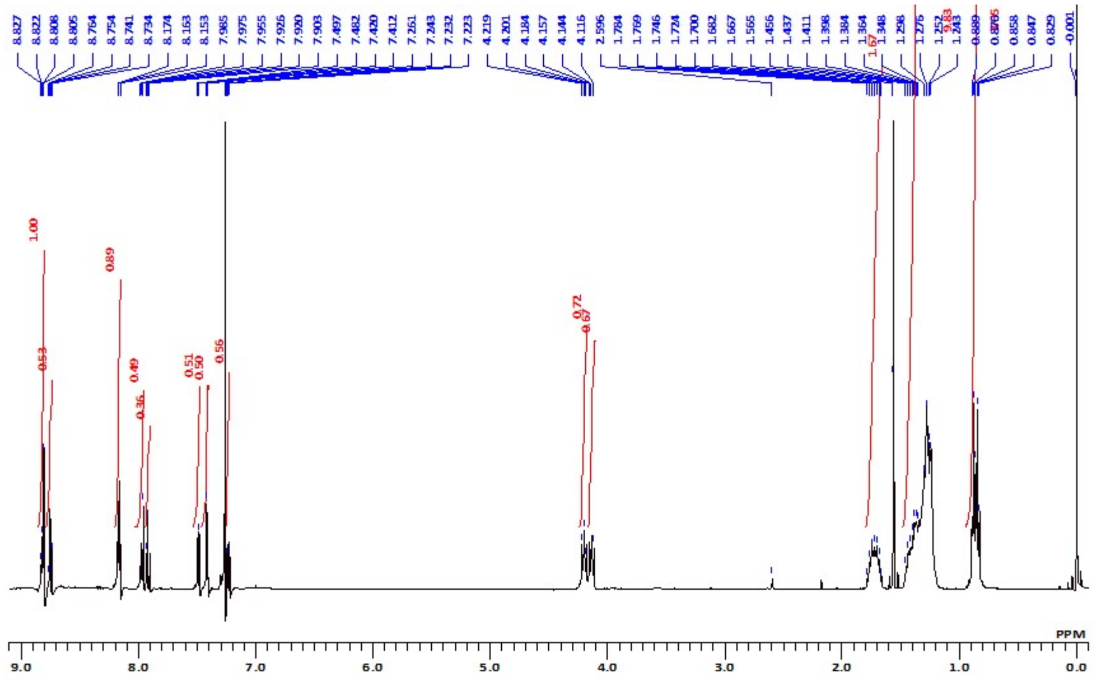

Figure S5 ^{1}H NMR spectrum of NDI-BTT in $CDCl_3$.

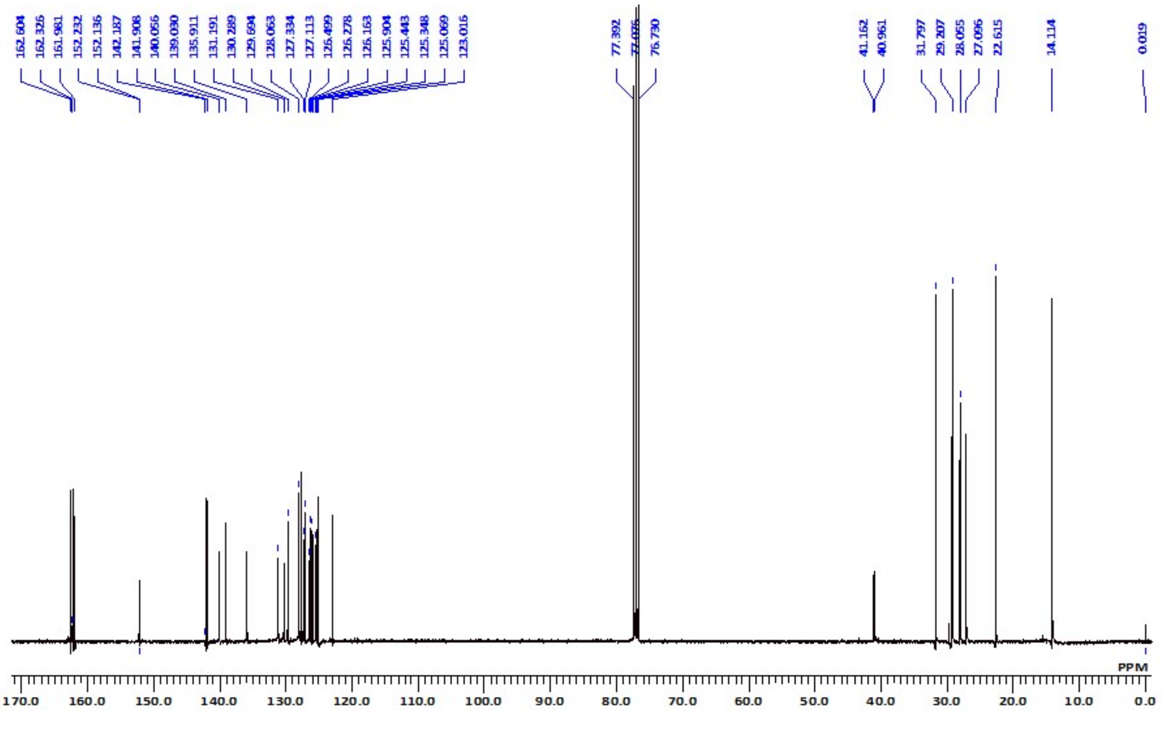

Figure S6 ^{13}C NMR spectrum of NDI-BTT in $CDCl_3$.

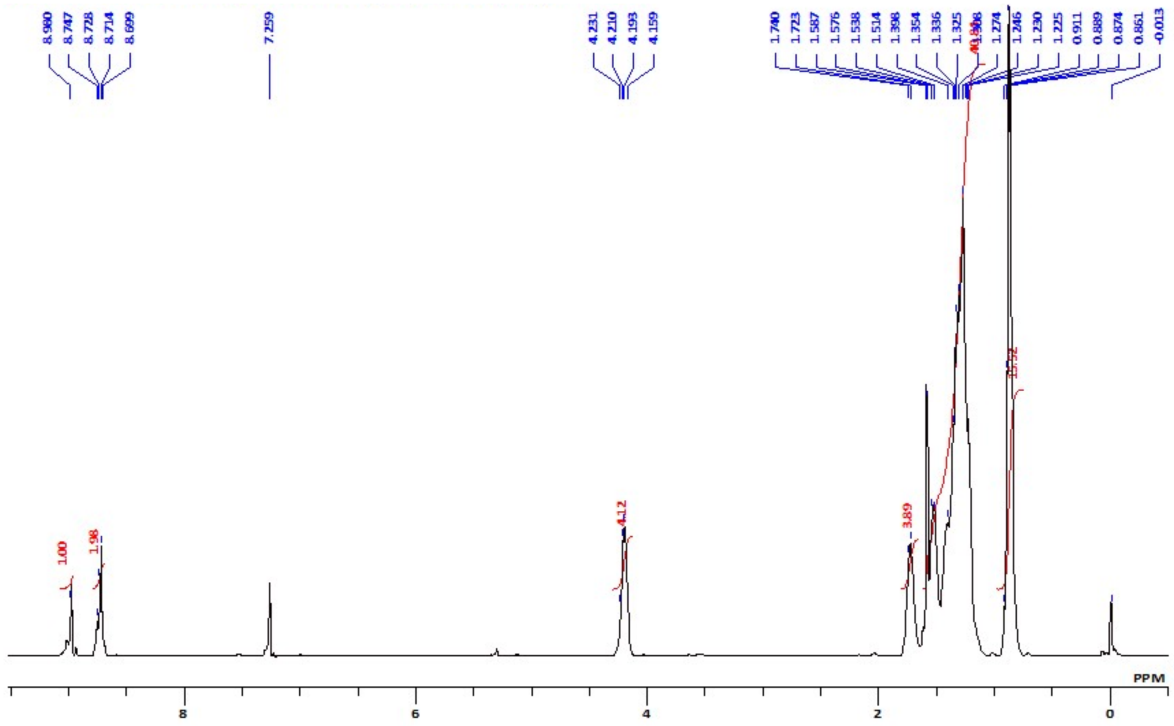

Figure S7 ^{1}H NMR spectrum of **3** in $CDCl_3$.

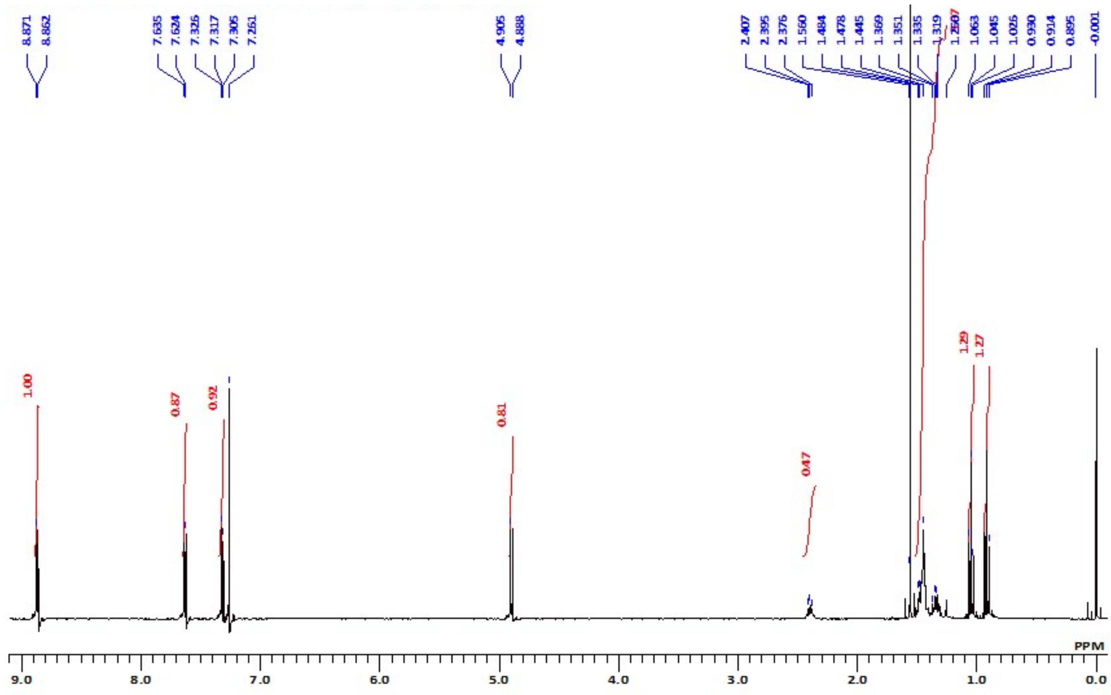

Figure S8 ^{1}H NMR spectrum of **7** in $CDCl_3$.

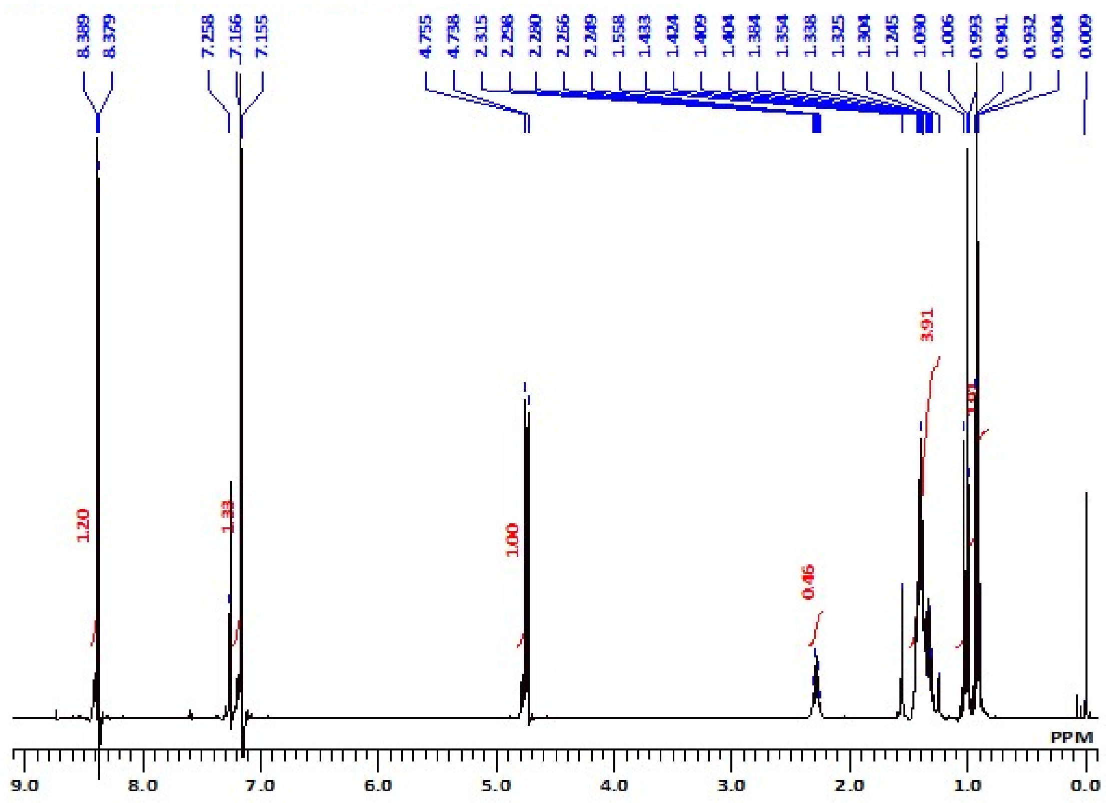

Figure S9 ^{1}H NMR spectrum of **4** in $CDCl_3$.

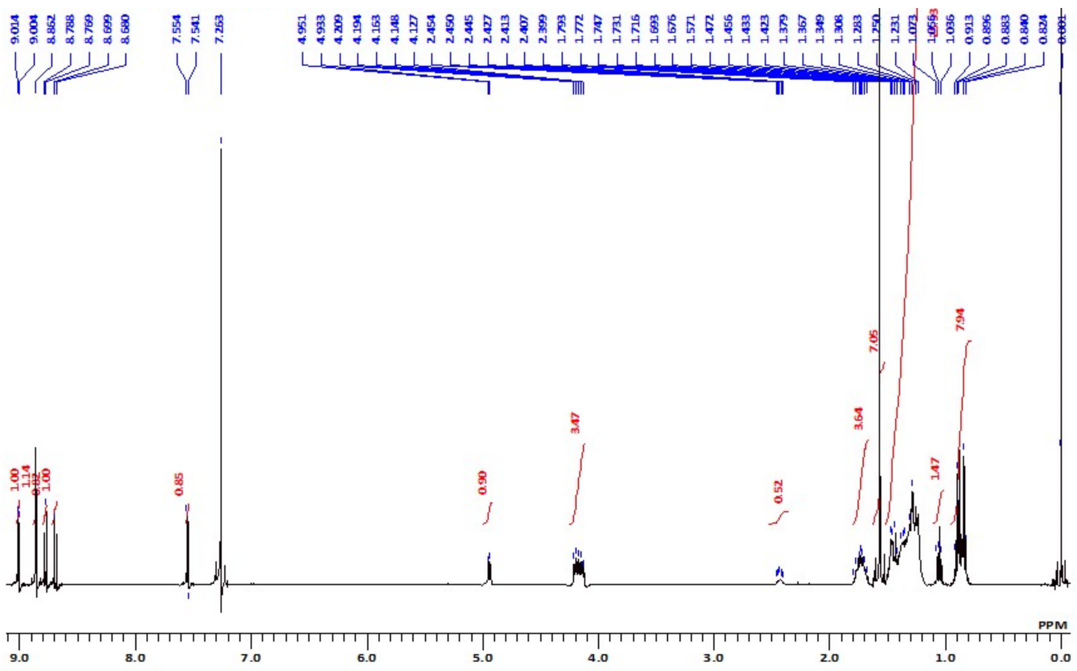

Figure S10 ^{1}H NMR spectrum of NDI-TBZT-NDI in $CDCl_3$.

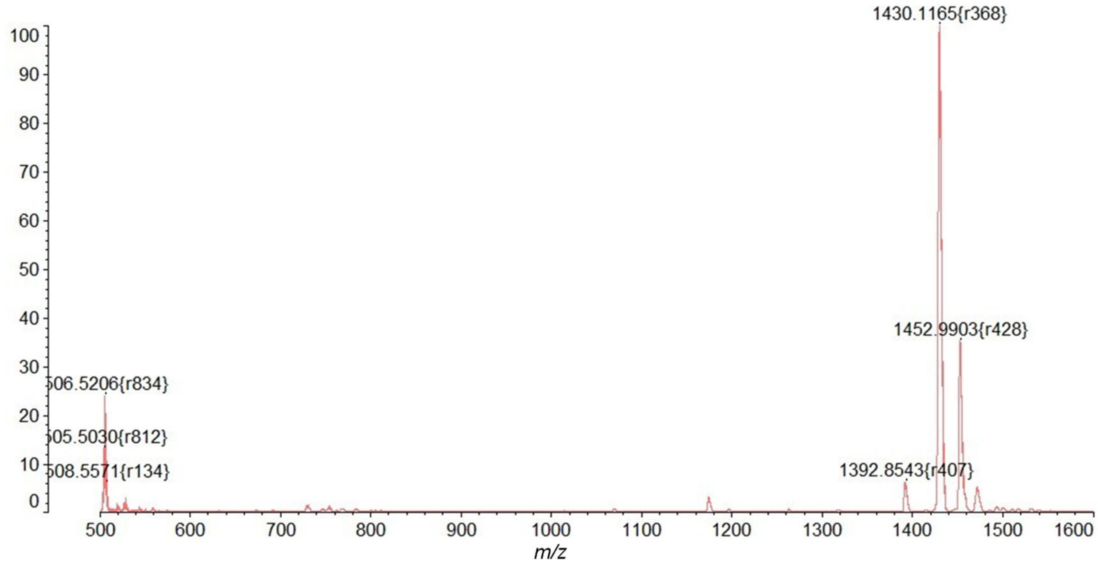

Figure S11 MALDI-TOF mass spectrum of NDI-TBZT-NDI (matrix: dithranol).

2. Supplementary Figures

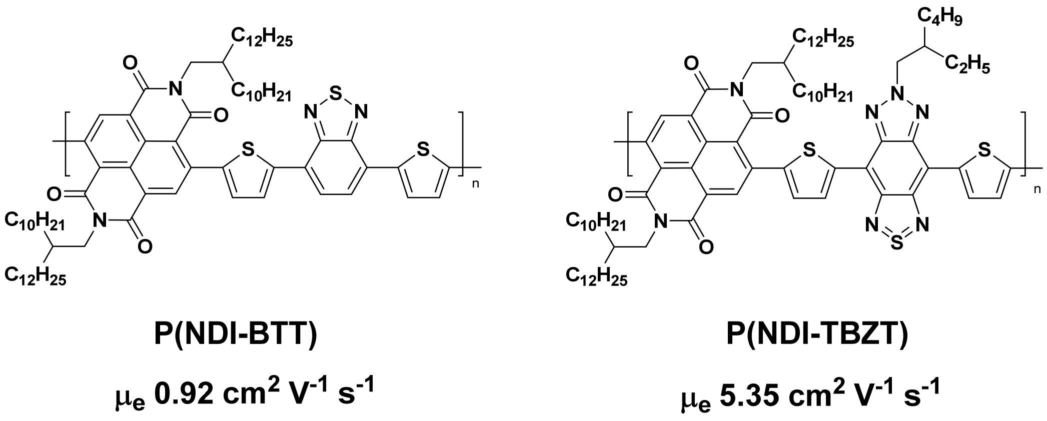

Figure S12 Previously reported D-A1-D-A2 type copolymers based on NDI and BT/TBZ acceptors. [S2]

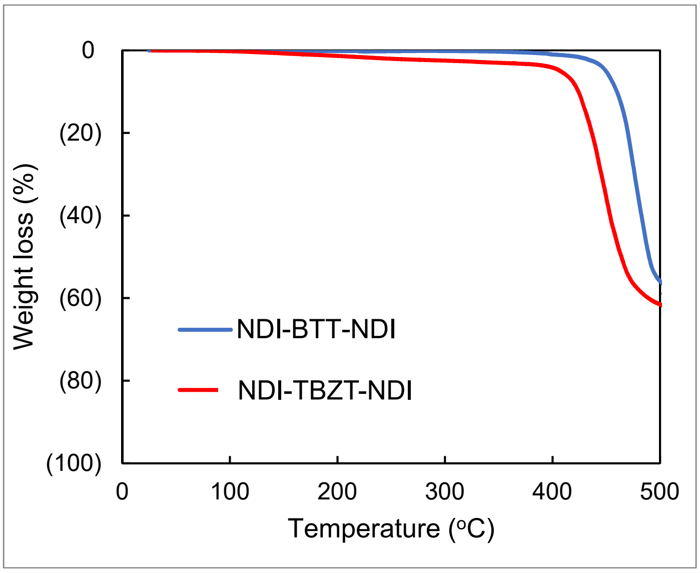

Figure S13 Thermogravimetric analysis (TGA) of NDI-BTT-NDI and NDI-TBZT-NDI under nitrogen flow at the heating rate of 10 °C min^{-1}.

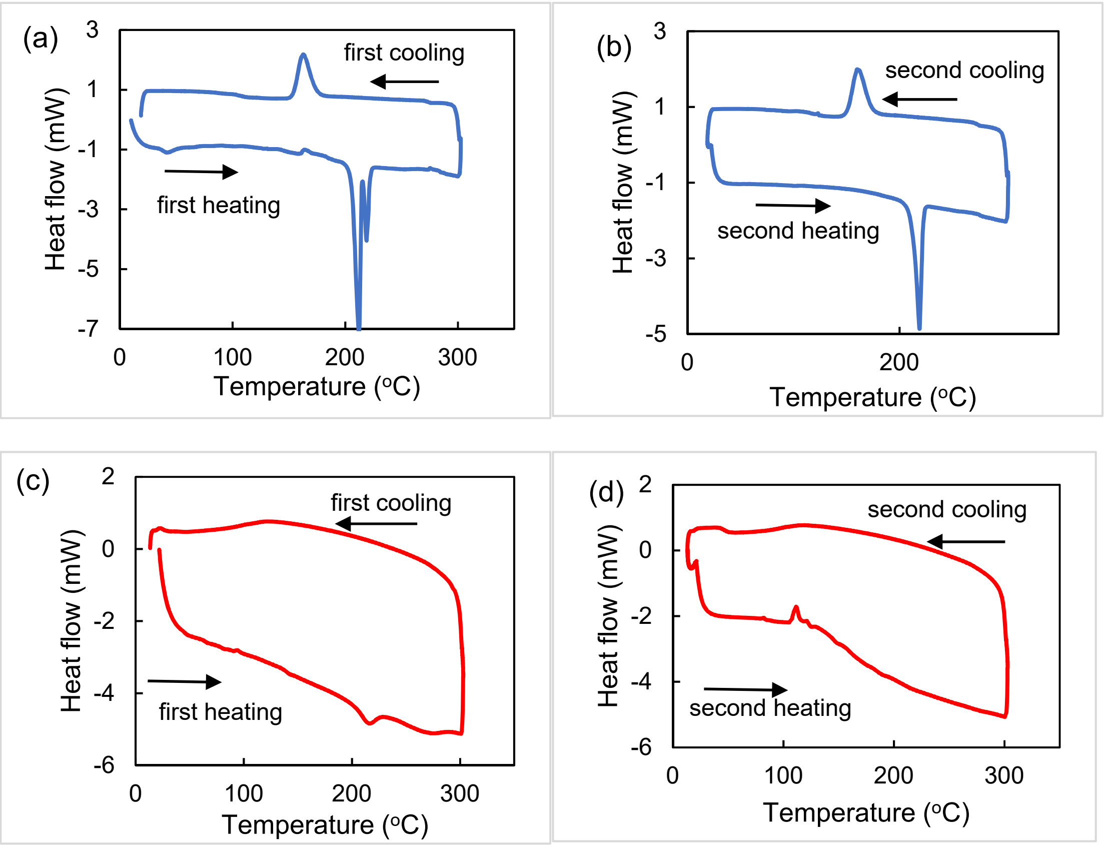

Figure S14 Differential scanning calorimetry (DSC) curves of (a,b) NDI-BTT-NDI and (c,d) NDI-TBZT-NDI under nitrogen flow (50 mL min^{-1}) at the scanning rate of 10 °C min^{-1}.

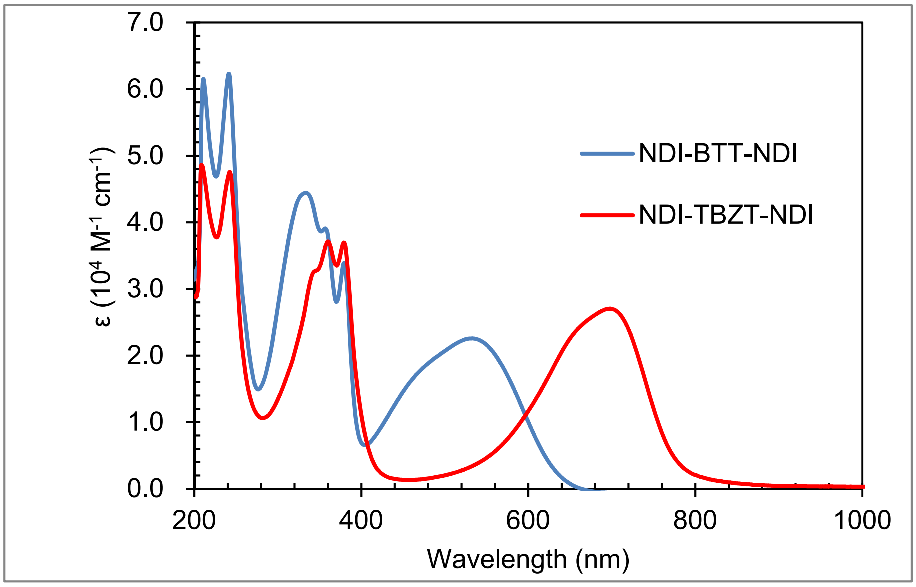

Figure S15 UV-vis-near IR absorption spectra of NDI-BTT-NDI and NDI-TBZT-NDI in $CHCl_3$, 10^{-5} M solution.

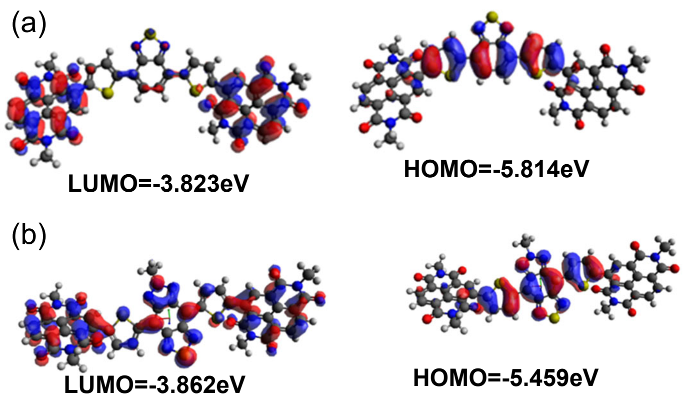

Figure S16 Optimized structures and HOMO and LUMO distributions of (a) NDI-BTT-NDI and (b) NDI-TBZT-NDI. Calculations were conducted at the DFT, B3LYP/6-311+G level.

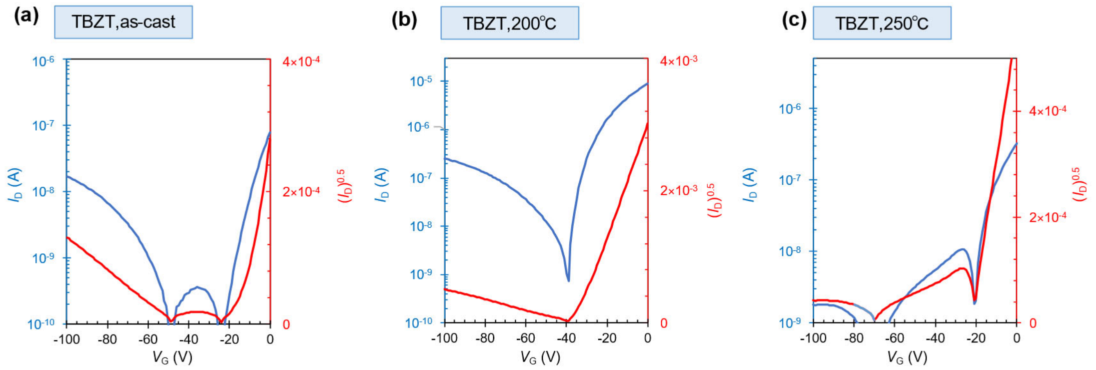

Figure S17 Organic field-effect transistor performances of transfer characteristics based on NDI-TBZT-NDI under p-type operation (a: as-cast, b: after annealing at 200 °C for 10 min, c: after annealing at 250 °C for 10 min), measured in vacuum (transistors have a channel length of L = 100 μm and a width of W = 1 mm).

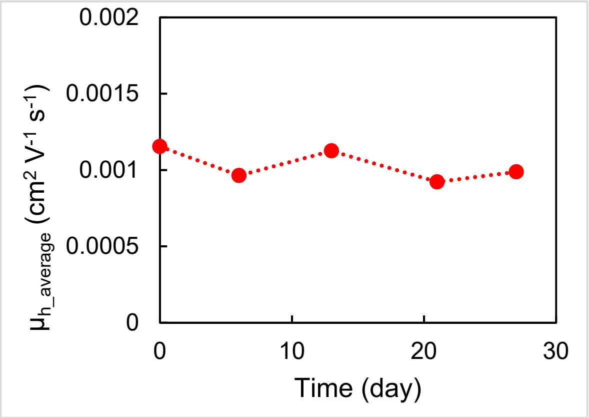

Figure S18 Air stability of the p-channel performance of the NDI-TBZT-NDI-based transistor.

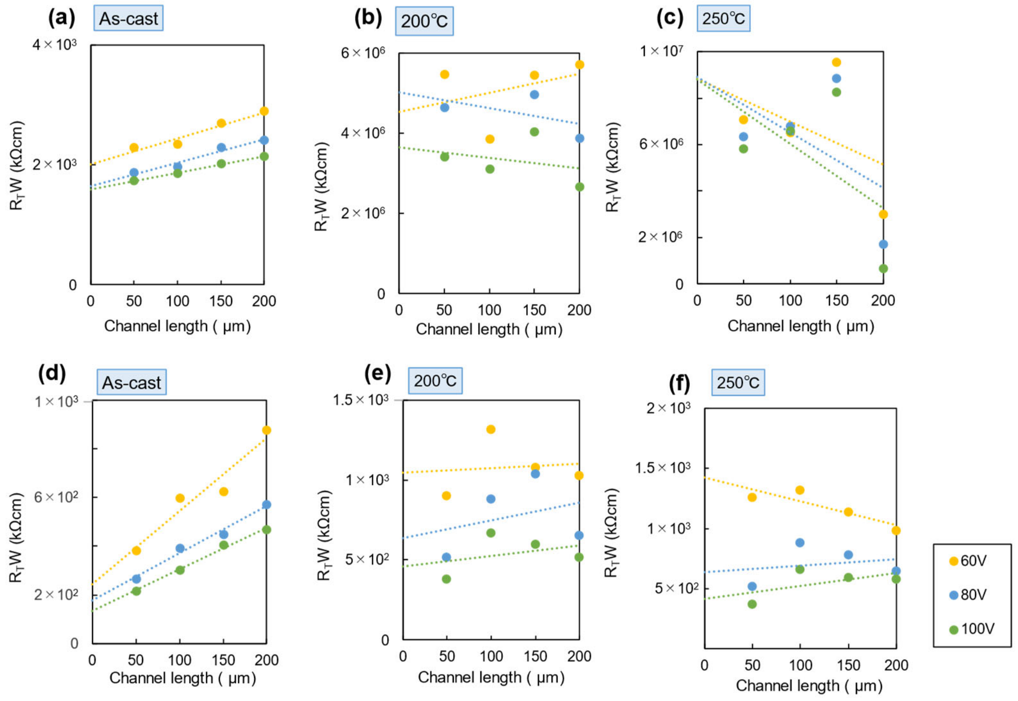

Figure S19 Channel length dependence of width-normalized total resistance (R_TW) with Au contact of OFETs based on (a)-(c) NDI-BTT-NDI and (d)-(f) NDI-TBZT-NDI.

Table S1 Surface free energy of the thin films of NDI-BTT-NDI and NDI-TBZT-NDI

	Annealing temp. (°C)	θ_{water} (°)	$\theta_{glycerin}$ (°)	γ (mN/m)
NDI-BTT-NDI	As cast	102.0	93.48	14.48
	200°C	105.2	94.21	15.46
	250°C	100.9	92.11	15.27
NDI-TBZT-NDI	As cast	104.0	92.53	16.52
	200°C	102.0	96.81	12.32
	250°C	96.18	101.9	17.36

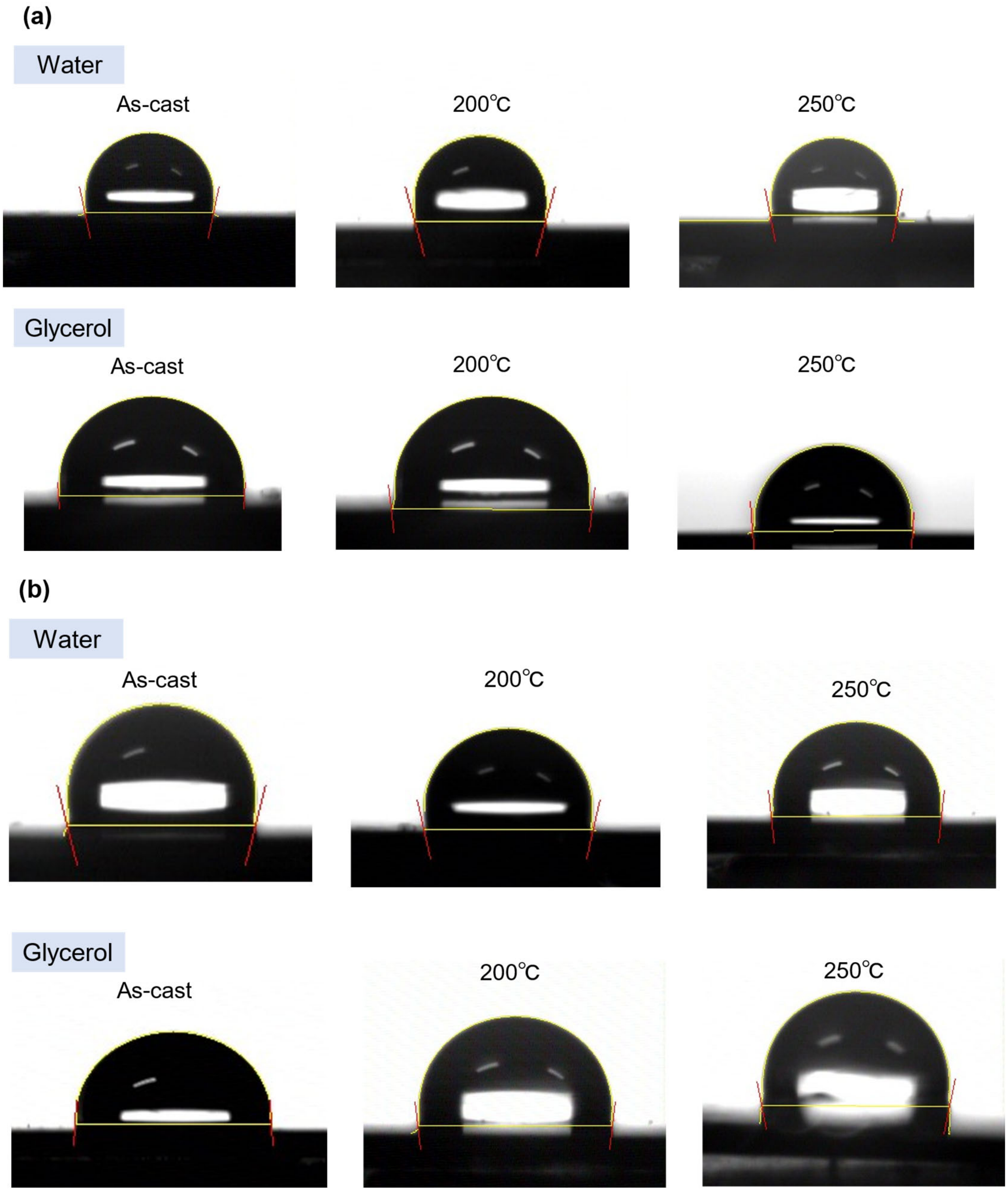

Figure S20 Contact angle measurements of thin film based (a) NDI-BTT-NDI and (b) NDI-TBZT-NDI.

Reference

[S1] W. Yue, A. Lv, J. Gao, W. Jiang, L. Hao, C. Li, Y. Li, L. E. Polander, S. Barlow, W. Hu, S. D. Motta, F. Negri, S. R. Marder, Z. Wang, *J. Am. Chem. Soc.* **2012**, *134*, 5770–5773.

[S2] Y. Wang, T. Hasegawa, H. Matsumoto, T. Mori, T. Michinobu, *Adv. Mater.* **2018**, *30*, 1707164.
